# Chromium(II)-catalyzed enantioselective arylation of ketones

**DOI:** 10.3762/bjoc.12.275

**Published:** 2016-12-19

**Authors:** Gang Wang, Shutao Sun, Ying Mao, Zhiyu Xie, Lei Liu

**Affiliations:** 1 Shenzhen Research Institute of Shandong University, Shenzhen 518057, China; 2 School of Pharmaceutical Sciences, Shandong University, Jinan 250012, China; 3 School of Chemistry and Chemical Engineering, Shandong University, Jinan 250100, China

**Keywords:** arylation, asymmetric catalysis, chromium, ketone, tertiary alcohol

## Abstract

The chromium-catalyzed enantioselective addition of carbo halides to carbonyl compounds is an important transformation in organic synthesis. However, the corresponding catalytic enantioselective arylation of ketones has not been reported to date. Herein, we report the first Cr-catalyzed enantioselective addition of aryl halides to both arylaliphatic and aliphatic ketones with high enantioselectivity in an intramolecular version, providing facile access to enantiopure tetrahydronaphthalen-1-ols and 2,3-dihydro-1*H*-inden-1-ols containing a tertiary alcohol.

## Introduction

Catalytic enantioselective carbon–carbon bond formation reactions have achieved enormous development during the last few decades as a consequence of the growing demand for enantiopure compounds in modern industry, especially the pharmaceutical industry. The chromium (Cr)-catalyzed enantioselective addition of carbo halides to carbonyl compounds is one of the most reliable methods in organic chemistry for chemoselective and structurally diverse synthesis [1–9]. To date, the Cr-catalyzed enantioselective carbonyl addition reactions mainly focused on allylation, propargylation, alkenylation and alkylation of aldehydes [10–11]. Since the first example of enantioselective allylation of aldehydes catalyzed by a Cr(II)–salen complex in 1999 by Cozzi and co-workers [12], several elegant catalytic enantioselective allylation and propargylation reactions have been developed by the groups of Nakada [13–14], Berkessel [15], Kishi [16], Sigman [17], Yamamoto [18], Guiry [19], Chen [20], Gade [21], White [22], and Zhang [23–25], respectively. The alkenylation and alkylation reactions were mainly explored by the Kishi group [26–30], and they established a toolbox approach to search for the specific ligand with a given substrate in the Cr-catalyzed process [28]. They successfully applied the method to the natural product total synthesis like halichondrin B and norhalichondrin B, and in the subsequent pharmaceutical study, finally leading to the discovery of the anticancer drug Eribulin [31–35]. However, to our knowledge, the Cr-catalyzed enantioselective arylation of carbonyl compounds has rarely been explored. On the other hand, most of the reactions focused on aldehyde components, while asymmetric addition to ketones remains a big challenge probably due to the decreased reactivity and selectivity [36–37]. A breakthrough was made by the Sigman group who reported the catalytic enantioselective addition of allylic bromides and propargyl halides to arylaliphatic ketones using oxazoline ligands with high enantioselectivity (up to 95% ee) [38–41]. After that, the Chen group also disclosed enantioselective allylation of ketones using spirocyclic chiral borate and chiral bipyridyl alcohol ligands with the ee value ranging from 27% to 97% [42–43]. However, as far as we know, a Cr-catalyzed enantioselective arylation of ketones has never been reported to date [44]. Tetrahydronaphthalen-1-ol bears a chiral tertiary alcohol center and is a common structural motif in numerous biologically active natural products and clinical drugs [45]. The method to prepare these compounds through intramolecular arylation of ketones would be highly desired. Herein, we report the first Cr-catalyzed enantioselective arylation of ketones in an intramolecular version.

## Results and Discussion

Initially, the Cr-catalyzed asymmetric intramolecular arylation of arylaliphatic ketone 5-(2-iodophenyl)pentan-2-one (**1a**) was selected as the model reaction for optimization employing Kishi’s oxazoline/sulfonamides as the chiral ligands. A series of oxazoline/sulfonamide ligands (**L1**–**L8**) were tested and the results were summarized in Table 1. Four subgroups of R^1^ were studied (entries 1–4, Table 1) and isopropyl substituted oxazoline proved to be the best ligand with a 42% ee. Afterwards, R^2^ (Table 1, entries 2, 5 and 6) and R^3^ (Table 1, entries 6–8) substituents were also examined, and **L8** bearing a methyl group in both R^2^ and R^3^ gave the best enantiocontrol. The solvent effect was then investigated, and 1,2-dimethoxyethane (DME) was identified to be the best choice (Table 1, entries 8–10). Lowering the reaction temperature was found to be beneficial for improving the enantioselectivity, and when the reaction was performed at −20 °C, expected **2a** was isolated in 81% yield with 97% ee (Table 1, entries 10–13). Aryl bromide proved to be an inferior coupling component, providing **2a** in 35% yield and 70% ee (Table 1, entry 14).

**Table 1:** Screening conditions of the catalytic enantioselective Cr-mediated arylation of ketone.^a^

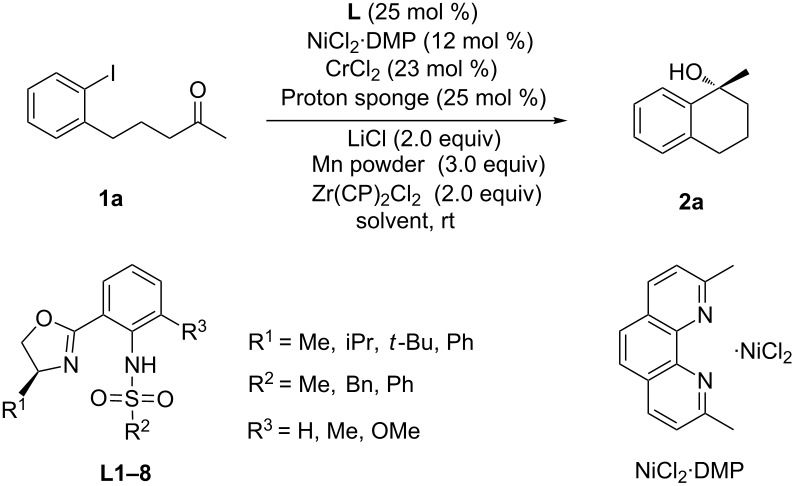							

entry	**L**	R^1^	R^2^	R^3^	Solvent	Yield (%)^a^	ee^b^

1	**L1**	Me	Ph	H	MeCN	43	28
2	**L2**	iPr	Ph	H	MeCN	50	42
3	**L3**	*t*-Bu	Ph	H	MeCN	47	25
4	**L4**	Ph	Ph	H	MeCN	51	21
5	**L5**	iPr	Bn	H	MeCN	12	37
6	**L6**	iPr	Me	H	MeCN	49	56
7	**L7**	iPr	Me	OMe	MeCN	75	75
8	**L8**	iPr	Me	Me	MeCN	85	82
9	**L8**	iPr	Me	Me	THF	93	81
10	**L8**	iPr	Me	Me	DME	90	86
11^c^	**L8**	iPr	Me	Me	DME	86	92
12^d^	**L8**	iPr	Me	Me	DME	81	97
13^e^	**L8**	iPr	Me	Me	DME	51	83
14^d,f^	**L8**	iPr	Me	Me	DME	35	70

^a^Yield of isolated product. ^b^Determined by HPLC analysis on a chiral column. ^c^Reaction at 0 °C for 24 h. ^d^Reaction at −20 °C for 24 h. ^e^Reaction at −40 °C for 24 h. ^f^Aryl bromide used instead of aryl iodide.

**Scheme 1:**
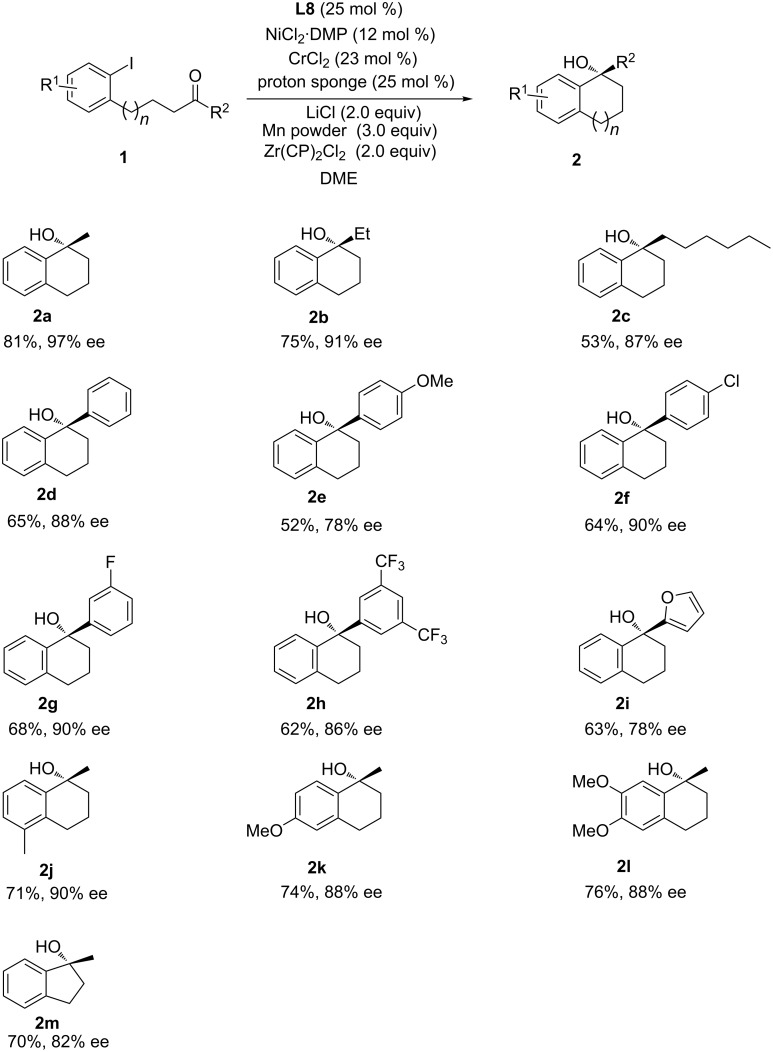
Scope of the catalytic enantioselective Cr-mediated arylation of ketones.

With the optimized conditions in hand, the scope of the ketone component was first explored (Scheme 1). Aliphatic ketones with (longer) alkyl chain such as ethyl (**1b**) and *n*-hexyl ketones (**1c**), were also tolerated albeit with slightly decreased yield and selectivity. The asymmetric arylation of various arylaliphatic ketones also went smoothly (**1d**–**h**). Phenyl ketone **1d** and ketones with electron-withdrawing groups in different substituent patterns gave the expected products with good enantiocontrol, while the enantioselectivity for ketone **1e** bearing an electron-donating group decreased. The mild process exhibited excellent functional group tolerance, with chloride (**2f**), fluoride (**2g**), and CF_3_ moieties (**2h**) well tolerated for further manipulation [46–47]. Heteroaryl ketones such as furan-substituted ketone (**1i**) were also suitable substrate, giving product **2i** in 78% ee. The scope of the aryl halide component was next explored (**1j**–**l**). Aryl halides bearing different substituent patterns were tolerated giving the tetrahydronaphthalen-1-ols with good ee values. When 4-(2-iodophenyl)butan-2-one (**1m**) was used, enantiopure indan-1-ol was obtained in 70% yield and 82% ee.

## Conclusion

In summary, we have developed the first Cr-catalyzed enantioselective arylation of ketones in an intramolecular version using oxazoline/sulfonamide **L8** as the catalyst. Both aliphatic and arylaliphatic ketones proceeded smoothly, providing corresponding tetrahydronaphthalen-1-ols bearing a tertiary alcohol center with good enantioselectivities (up to 97% ee).

## Experimental

**General procedure for the chromium(II) catalyzed enantioselective arylation of ketones**: The solution of **L8** (0.25 equiv, 0.025 mmol), proton sponge (0.25 equiv, 0.025 mmol) and CrCl_2_ (0.23 equiv, 0.023 mmol) in DME (1.0 mL) was stirred at room temperature in a glove-box for 1 h. Then the substrate **1** (1.0 equiv, 0.1 mmol), LiCl (2.0 equiv, 0.2 mmol), Mn powder (3.0 equiv, 0.3 mmol), NiCl_2_·DMP (0.12 equiv, 0.012 mmol) and Zr(CP)_2_Cl_2_ (2.0 equiv, 0.2 mmol) were added successively and the mixture was stirred at indicated temperature for 24 h. After that, the mixture was filtered through a short pad of celite and purified by flash chromatography using silica gel or alumina (200–300 mesh) to give the product **2**.

## Supporting Information

File 1 Experimental procedures, analytical data for products, copies of NMR spectra and HPLC chromatograms.
