# Supplementary material for: Chromium(II)-catalyzed enantioselective arylation of ketones
Source: Beilstein J Org Chem. 2016 Dec 19;12:2771–5. doi: 10.3762/bjoc.12.275 (PMC5238525; doi:10.3762/bjoc.12.275)
Supplement: File 1 — Experimental procedures, analytical data for products, copies of NMR spectra and HPLC chromatograms. [file Beilstein_J_Org_Chem-12-2771-s001.pdf]

Supporting Information

for

Chromium(II)-catalyzed enantioselective arylation of ketones

Gang Wang,[1,3] Shutao Sun,[3] Ying Mao,[3] Zhiyu Xie[3] and Lei Liu*[1,2,3]

Address: [1]Shenzhen Research Institute of Shandong University, Shenzhen 518057, China; [2]School of Chemistry and Chemical Engineering, Shandong University, Jinan 250100, China, and [3]School of Pharmaceutical Sciences, Shandong University, Jinan 250012, China

Email: Lei Liu* - leiliu@sdu.edu.cn

*Corresponding author

Experimental procedures, analytical data for products, copies of NMR spectra and HPLC chromatograms

Contents

General methods--S2

Analytical data for products--S3

References--S9

^{1}H and ^{13}C NMR Spectra---S10

HPLC data--S22

General methods

Proton (^{1}H NMR) and carbon (^{13}C NMR) nuclear magnetic resonance spectra were recorded at 400 MHz and 100 MHz, respectively. The chemical shifts are given in parts per million (ppm) on the delta (δ) scale. The solvent peak was used as a reference value, for ^{1}H NMR: $CDCl_3$ δ 7.27, DMSO-d_6 δ 2.50; for ^{13}C NMR: $CDCl_3$ at 77.23 ppm, DMSO-d_6 at 39.51 ppm. Analytical TLC was performed on precoated silica gel GF254 plates. Column chromatography was carried out on silica gel or alumina (200–300 mesh). HRMS were carried out on an Orbitrap analyzer. Optical rotations were measured using a 1.0 mL cell with a 10 cm path length on ANTON PAAR MCP 200 polarimeter and concentrations (c) were reported in g×(100 mL)$^{-1}$. Enantiomeric excesses were determined by HPLC using a Daicel Chiralpak AD-H, AS-H or OD-H column with hexane/iPrOH as the eluent. Reaction vessels were oven-dried, and all reactions were conducted in a glove-box. $CrCl_2$, LiCl, proton sponge, Mn powder and super-dry solvent MeCN, THF, DME were purchased from Aldrich and used as supplied. The sulfonamide ligands were synthesized using the same method as literature reported [5].

Analytical data for products

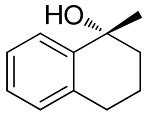

(*R*)-1-Methyl-1,2,3,4-tetrahydronaphthalen-1-ol (2a)

It was prepared following the general procedure at -20°C. 13.1mg, 81% yield, colorless oil. ^{1}H NMR (400 MHz, $CDCl_3$) δ 7.47 (d, *J* = 7.6 Hz, 1H), 7.16–7.02 (m, 2H), 6.96 (d, *J* = 7.4 Hz, 1H), 2.77–2.58 (m, 2H), 1.91 (s, 1H), 1.86–1.66 (m, 4H), 1.44 (s, 3H); ^{13}C NMR (101 MHz, $CDCl_3$) δ 143.0, 136.4, 128.9, 127.2, 126.5, 126.5, 70.7, 39.9, 30.9, 30.0, 20.5. These data are consistent with literature report [1]. HPLC: the ee value was determined by HPLC analysis (Chiralcel AD-H, *i*-PrOH/Hexane = 10/90, 0.5 mL/min, 265 nm), retention time: t_{minor} = 11.867 min, t_{major} = 12.950 min, ee = 97%; $[\alpha]_D^{20}$= – 28.3 (c = 0.51, $CHCl_3$)[lit.:[2] $[\alpha]_D^{20}$= – 29.5 (c 3.8, $CHCl_3$), and the absolute configuration of the chiral center can also be assigned as *R*]

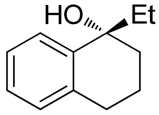

(*R*)-1-Ethyl-1,2,3,4-tetrahydronaphthalen-1-ol (2b)

It was prepared following the general procedure at -20°C. 13.2 mg, 75% yield, colorless oil. ^{1}H NMR (400 MHz, $CDCl_3$) δ 7.45 (d, *J* = 7.5 Hz, 1H), 7.17–7.06 (m, 2H), 7.01 (d, *J* = 7.4 Hz, 1H), 2.79–2.59 (m, 2H), 2.00–1.90 (m, 1H), 1.86–1.69 (m, 5H), 1.64 (s, 1H), 0.82 (t, *J* = 7.5 Hz, 3H); ^{13}C NMR (101 MHz, $CDCl_3$) δ 142.5, 137.1, 129.1, 127.2, 126.5, 72.9, 35.6, 35.1, 30.2, 19.9, 8.8. These data are consistent with literature report [1]. HPLC: the ee value was determined by HPLC analysis (Chiralcel AD-H, *i*-PrOH/Hexane = 10/90, 1.0 mL/min, 220 nm), retention time: t_{minor} = 6.174 min, t_{major} = 6.988 min, ee = 91%; $[\alpha]_D^{20}$ = – 0.98 (c = 0.18, MeOH).

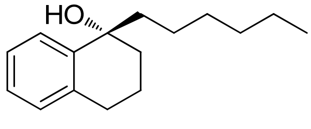

(*R*)-1-Hexyl-1,2,3,4-tetrahydronaphthalen-1-ol (2c)

It was prepared following the general procedure at 0°C and purified on basic alumina. 12.3 mg, 53% yield, colorless oil. ^{1}H NMR (400 MHz, DMSO-*d*$_6$) δ 6.68 (dd, *J* = 7.7,

1.2 Hz, 1H), 6.37–6.26 (m, 2H), 6.25–6.22 (m, 1H), 2.04–1.85 (m, 2H), 1.24–0.88 (m, 6H), 0.55–0.38 (m, 8H), 0.07 (dd, J = 8.5, 5.0 Hz, 3H); ^{13}C NMR (101 MHz, DMSO-d_6) δ 134.0, 128.5, 120.2, 118.2, 118.1, 117.4, 63.7, 34.4, 27.5, 23.5, 21.5, 21.5, 15.8, 14.1, 11.3, 4.9. HRMS (EI) *m/z* $[M + H]^+$calculated for $C_{16}H_{25}O$: 233.1900, found 233.1905. HPLC: the ee value was determined by HPLC analysis (Chiralcel AD-H, *i*-PrOH/Hexane = 3/97, 0.5 mL/min, 220 nm), retention time: t_{minor} = 15.491 min, t_{major} = 17.776 min, ee =87%; $[\alpha]_D^{25}$= – 4.8 (c = 0.16, MeOH).

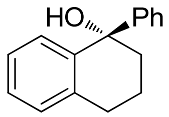

(*S*)-1-Phenyl-1,2,3,4-tetrahydronaphthalen-1-ol (2d)

It was prepared following the general procedure at 0°C. 14.5 mg, 65% yield, colorless oil. ^{1}H NMR (400 MHz, $CDCl_3$) δ 7.39–7.30 (m, 4H), 7.29–7.12 (m, 4H), 7.12–7.05 (m, 1H), 2.97–2.88 (m, 2H), 2.24–2.14 (m, 3H), 2.10–1.99 (m, 1H), 1.88–1.77 (m, 1H); ^{13}C NMR (101 MHz, $CDCl_3$) δ 149.2, 142.3, 137.8, 129.1, 129.1, 128.0, 127.7, 126.8, 126.7, 126.6, 75.6, 41.7, 30.1, 19.9. These data are consistent with literature report [1]. HPLC: the ee value was determined by HPLC analysis (Chiralcel AD-H, *i*-PrOH/Hexane = 10/90, 1.0 mL/min, 260 nm), retention time: t_{minor} = 7.394 min, t_{major} = 8.397 min, ee = 88%; $[\alpha]_D^{20}$ = – 28.1 (c = 0.60, $CHCl_3$).

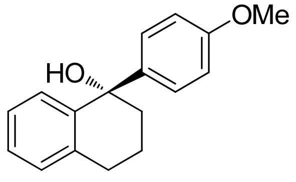

(*S*)-1-(4-Methoxyphenyl)-1,2,3,4-tetrahydronaphthalen-1-ol (2e)

It was prepared following the general procedure at 0°C and purified on basic alumina. 13.2 mg, 52% yield, colorless oil. ^{1}H NMR (400 MHz, DMSO-d_6) δ 7.20–6.99 (m, 6H), 6.82 (d, J = 8.8 Hz, 2H), 5.45 (s, 1H), 3.72 (s, 3H), 2.80 (t, J = 6.2 Hz, 2H), 2.09–1.95 (m, 2H), 1.94–1.82 (m, 1H), 1.63–1.53 (m, 1H); ^{13}C NMR (101 MHz, DMSO-d_6) δ 158.0, 143.5, 142.7, 137.2, 129.6, 128.6, 127.9, 127.0, 126.0, 113.2, 73.9, 55.4, 41.5, 29.8, 19.7. These data are consistent with literature report [3]. HPLC: the ee value was determined by HPLC analysis (Chiralcel OD-H, *i*-PrOH/Hexane =

10/90, 1.0 mL/min, 275 nm), retention time: t_{minor} = 6.900 min, t_{major} = 10.375 min, ee = 78%; $[\alpha]_D^{20}$ = – 23.3 (c = 0.45, $CHCl_3$).

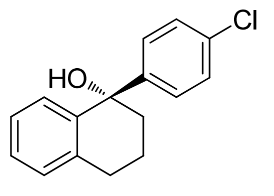

(*S*)-1-(4-Chlorophenyl)-1,2,3,4-tetrahydronaphthalen-1-ol (2f)

It was prepared following the general procedure at 0°C. 16.5 mg, 64% yield, colorless oil. ^1H NMR (400 MHz, $CDCl_3$) δ 7.34–7.27 (m,4H), 7.27–7.20 (m, 2H), 7.19–7.12 (m, 1H), 7.05 (d, *J* = 7.7 Hz, 1H), 2.94 (t, *J* = 5.9 Hz, 2H), 2.45 (s, 1H), 2.20–1.97 (m, 3H), 1.89–1.78 (m, 1H); ^{13}C NMR (101 MHz, $CDCl_3$) δ 147.7, 141.6, 137.7, 132.5, 129.1, 129.0, 128.1, 127.9, 127.8, 126.7, 75.2, 41.5, 29.9, 19.7. These data are consistent with literature report [4]. HPLC: the ee value was determined by HPLC analysis (Chiralcel OD-H, *i*-PrOH/Hexane = 10/90, 1.0 mL/min, 265 nm), retention time: t_{minor} = 5.288 min, t_{major} = 7.797 min, ee = 90%; $[\alpha]_D^{20}$ = – 26.9 (c = 0.33, $CHCl_3$).

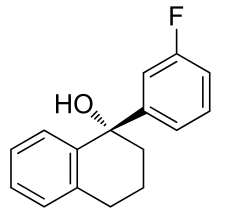

(*S*)-1-(3-Fluorophenyl)-1,2,3,4-tetrahydronaphthalen-1-ol (2g)

It was prepared following the general procedure at 0°C. 16.4 mg, 68% yield, colorless oil. ^1H NMR (400 MHz, $CDCl_3$) δ 7.34–7.14 (m, 5H), 7.13–7.05 (m, 2H), 7.01–6.95 (m, 1H), 2.95 (t, *J* = 6.3 Hz, 2H), 2.50 (s, 1H), 2.23–1.98 (m, 3H), 1.91–1.81 (m, 1H); ^{13}C NMR (101 MHz, $CDCl_3$) δ 163.9, 161.5, 152.1, 152.0, 141.5, 137.7, 129.3, 129.2, 129.1, 129.0, 127.8, 126.6, 122.3, 122.3, 113.9, 113.6, 113.6, 113.4, 75.2, 75.2, 41.4, 29.9, 19.6; HRMS (EI) *m/z* $[M + H]^+$ calculated for $C_{16}H_{16}FO$: 243.1180, found 243.1177. HPLC: the ee value was determined by HPLC analysis (Chiralcel OD-H,

i-PrOH/Hexane = 10/90, 1.0 mL/min, 263 nm), retention time: t_{minor} = 5.183 min, t_{major} = 6.942 min, ee = 90%; $[\alpha]_D^{20}$ = – 29.5 (c = 0.60, $CHCl_3$).

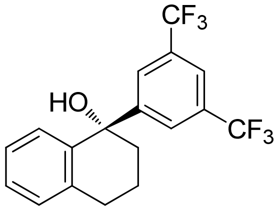

(*S*)-1-(3,5-Bis(trifluoromethyl)phenyl)-1,2,3,4-tetrahydronaphthalen-1-ol (2h)

It was prepared following the general procedure at 0°C. 22.3 mg, 62% yield, colorless oil. ^{1}H NMR (400 MHz, $CDCl_3$) δ 7.94 (s, 2H), 7.87 (s, 1H), 7.33–7.23 (m, 2H), 7.20–7.14 (m, 1H), 6.94 (d, *J* = 7.8 Hz, 1H), 3.00 (s, 2H), 2.74–2.67 (m, 1H), 2.24–2.05 (m, 3H), 1.95–1.83 (m, 1H); ^{13}C NMR (101 MHz, $CDCl_3$) δ 152.2, 140.5, 138.1, 131.8, 131.5, 131.2, 130.9, 129.6, 129.1, 128.5, 127.8, 127.1, 127.0, 126.9, 125.1, 122.4, 121.0, 121.0, 120.9, 120.9, 120.9, 119.7, 75.3, 41.7, 29.9, 19.6; HRMS (EI) *m/z* $[M + H]^+$ calculated for $C_{18}H_{15}F_6O$: 361.1022, found 361.1019. HPLC: the ee value was determined by HPLC analysis (Chiralcel OD-H, *i*-PrOH/Hexane = 10/90, 0.5 mL/min, 233 nm), retention time: t_{minor} = 9.764 min, t_{major} = 11.223 min, ee = 86%; $[\alpha]_D^{20}$ = – 25.6 (c = 0.37, $CHCl_3$).

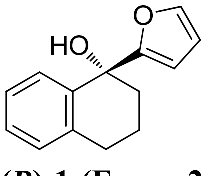

(*R*)-1-(Furan-2-yl)-1,2,3,4-tetrahydronaphthalen-1-ol (2i)

It was prepared following the general procedure at 0°C. 13.5 mg, 63% yield, colorless oil. ^{1}H NMR (400 MHz, $CDCl_3$) δ 7.49–7.37 (m, 2H), 7.30–7.16 (m, 3H), 6.33 (dd, *J* = 3.1, 1.8 Hz, 1H), 6.06 (d, *J* = 3.1 Hz, 1H), 2.96–2.81 (m, 2H), 2.76 (s, 1H), 2.52 (ddd, *J* = 12.7, 8.5, 2.8 Hz, 1H), 2.14–1.96 (m, 2H), 1.82–1.71 (m, 1H); ^{13}C NMR (101 MHz, $CDCl_3$) δ 159.2, 142.0, 139.2, 137.2, 129.0, 128.0, 127.9, 126.2, 110.0, 107.8, 72.1, 37.2, 29.5, 19.7; HRMS (EI) *m/z* $[M + H]^+$ calculated for $C_{14}H_{15}O_2$: 215.1067, found 215.1066. HPLC: the ee value was determined by HPLC analysis

(Chiralcel OD-H, *i*-PrOH/Hexane = 10/90, 1.0 mL/min, 233 nm), retention time: t_{minor} = 8.248 min, t_{major} = 10.407 min, ee = 78%; $[\alpha]_D^{20}$ = – 6.8 (c = 0.10, $CHCl_3$).

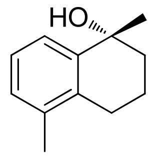

(*R*)-1,5-Dimethyl-1,2,3,4-tetrahydronaphthalen-1-ol (2j)

It was prepared following the general procedure at -20°C. 12.5 mg, 71% yield, colorless oil. ^1H NMR (400 MHz, $CDCl_3$) δ 7.50 (d, *J* = 7.8 Hz, 1H), 7.16 (t, *J* = 7.6 Hz, 1H), 7.08 (d, *J* = 7.3 Hz, 1H), 2.66 (t, *J* = 6.4 Hz, 2H), 2.24 (s, 3H), 2.04–1.82 (m, 4H), 1.77 (s, 1H), 1.58 (s, 3H); ^{13}C NMR (101 MHz, $CDCl_3$) δ 143.3, 136.4, 135.0, 128.8, 126.1, 124.1, 71.0, 39.5, 31.0, 27.2, 20.3, 20.1; HRMS (EI) *m/z* $[M + H]^+$ calculated for $C_{12}H_{17}O$: 177.1274, found 177.1277. HPLC: the ee value was determined by HPLC analysis (Chiralcel AS-H, *i*-PrOH/Hexane = 3/97, 1.0 mL/min, 220 nm), retention time: t_{minor} = 6.824 min, t_{major} = 7.878 min, ee = 90%; $[\alpha]_D^{20}$ = – 23.3 (c = 0.31, $CHCl_3$).

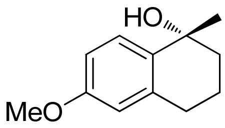

(*R*)-6-Methoxy-1-methyl-1,2,3,4-tetrahydronaphthalen-1-ol (2k)

It was prepared following the general procedure at -20°C and purified on basic alumina. 14.2 mg, 74% yield, colorless oil. ^1H NMR (400 MHz, DMSO-d_6) δ 7.42 (d, *J* = 8.6 Hz, 1H), 6.73 (dd, *J* = 8.6, 2.7 Hz, 1H), 6.56 (d, *J* = 2.5 Hz, 1H), 4.70 (s, 1H), 3.70 (s, 3H), 2.67 (dd, *J* = 11.8, 6.0 Hz, 2H), 1.91–1.62 (m, 4H), 1.36 (s, 3H); ^{13}C NMR (101 MHz, DMSO-d_6) δ 158.0, 137.4, 137.0, 128.3, 112.7, 68.9, 55.4, 39.9, 32.0, 30.3, 20.6. These data are consistent with literature report [1]. HPLC: the ee value was determined by HPLC analysis (Chiralcel AS-H, *i*-PrOH/Hexane = 10/90, 1.0 mL/min, 265 nm), retention time: t_{minor} = 6.192 min, t_{major} = 7.803 min, ee = 88%; $[\alpha]_D^{20}$ = – 11.5 (c = 0.20, $CHCl_3$).

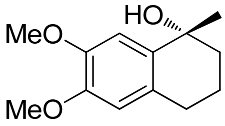

(*R*)-6,7-Dimethoxy-1-methyl-1,2,3,4-tetrahydronaphthalen-1-ol (2l)

It was prepared following the general procedure. 16.9 mg at -20°C, 76% yield, colorless oil. ^{1}H NMR (400 MHz, $CDCl_3$) δ 7.09 (s, 1H), 6.54 (s, 1H), 3.89 (s, 3H), 3.85 (s, 3H), 2.78–2.64 (m, 2H), 1.97–1.79 (m, 4H), 1.76 (s, 1H), 1.55 (s, 3H); ^{13}C NMR (101 MHz, $CDCl_3$) δ 148.4, 147.9, 135.0, 128.8, 111.3, 109.3, 70.8, 56.2, 56.0, 40.2, 30.9, 29.9, 20.9; HRMS (EI) *m/z* $[M + H]^+$ calculated for $C_{13}H_{19}O_3$: 223.1329, found 223.1330. HPLC: the ee value was determined by HPLC analysis (Chiralcel AD-H, *i*-PrOH/Hexane = 10/90, 1.0 mL/min, 265 nm), retention time: t_{minor} = 21.005 min, t_{major} = 19.301 min, ee = 88%; $[\alpha]_D^{20}$ = – 16.3 (c = 0.12, $CHCl_3$).

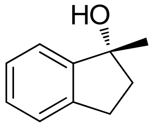

(*R*)-1-Methyl-2,3-dihydro-1H-inden-1-ol (2m)

It was prepared following the general procedure. 16.9 mg at -20°C, 76% yield, colorless oil.^{1}H NMR (400 MHz, $CDCl_3$) δ 7.43–7.34 (m, 1H), 7.30–7.21 (m, 3H), 3.12–2.98 (m, 1H), 2.90–2.79 (m, 2H), 2.35–2.14 (m, 2H), 1.89 (s, 1H), 1.59 (s, 3H); ^{13}C NMR (101 MHz, $CDCl_3$) δ 148.5, 142.8, 128.4, 127.0, 125.1, 122.4, 81.4, 42.5, 29.6, 27.5. These data are consistent with literature report [6]. HPLC: the ee value was determined by HPLC analysis (Chiralcel AD-H, *i*-PrOH/Hexane = 10/90, 0.5 mL/min, 273 nm), retention time: t_{minor} = 12.148 min, t_{major} = 13.191 min, ee = 82%.

References

1. Kelley, B. T.; Walters, J. C.; Wengryniuk, S. E. *Org. Lett.*, **2016**, *18* (8), 1896–1899

2. Jaouen, G.; Meyer, A. *J. Am. Chem. Soc.*, **1975**, *97*(16), 4667–4672.

3. Liao, Y.-X.; Xing, C.-H.; Hu, Q.-S. *Org. Lett.*, **2012**, *14*(6), 1544–1547

4. Qu, B.; Samankumara, L. P.; Ma, S.; Fandrick, K. R.; Desrosiers, J.-N.; Rodriguez, S.; Li, Z.; Haddad, N.; Han, Z. S.; McKellop, K.; Pennino, S.; Grinberg, N.; Gonnella, N. C.; Song, J. J.; Senanayake, C. H. *Angew. Chem., Int. Ed.*, **2014**, *53*(52), 14428–14432

5. Wan, Z.-K.; Choi, H.; Kang, F.-A.; Nakajima, K.; Demeke, D.; Kishi, Y. *Org. Lett.* **2002**, 4, 4431

6. Devrim Özdemirhan, Serdar Sezer, Yasemin Sönmez *Tetrahedron: Asymmetry* **2008**, 19, 2717–2720

^{1}H and ^{13}C NMR spectra

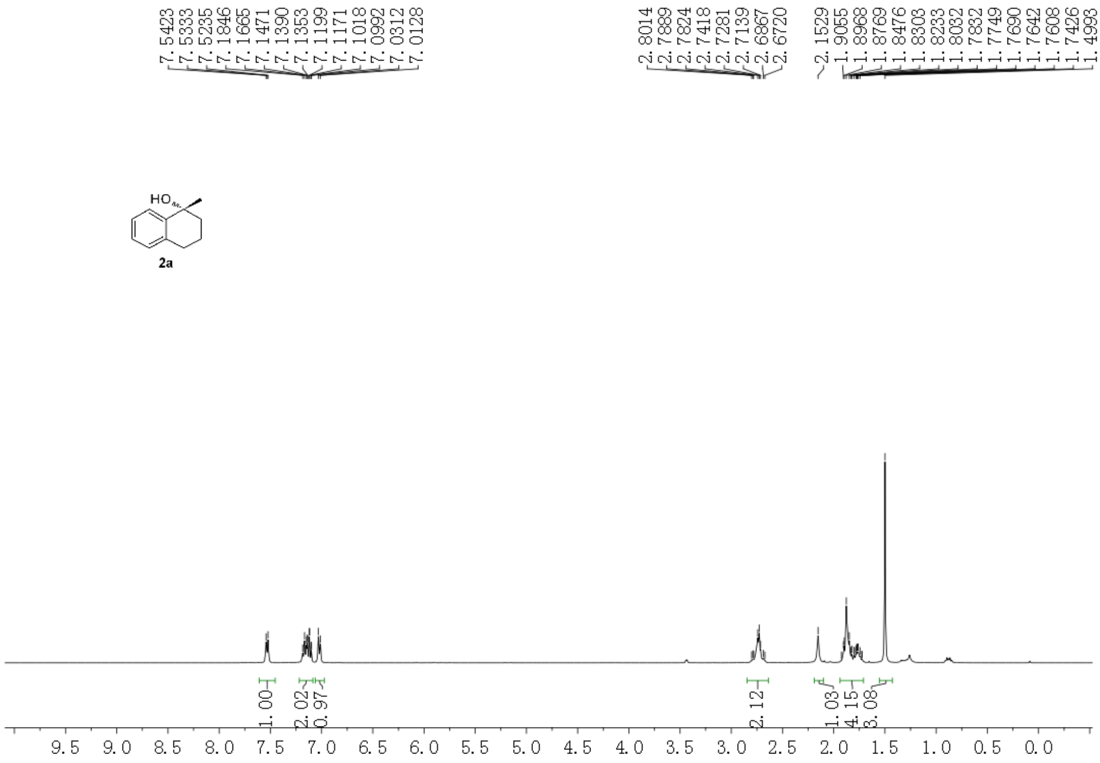

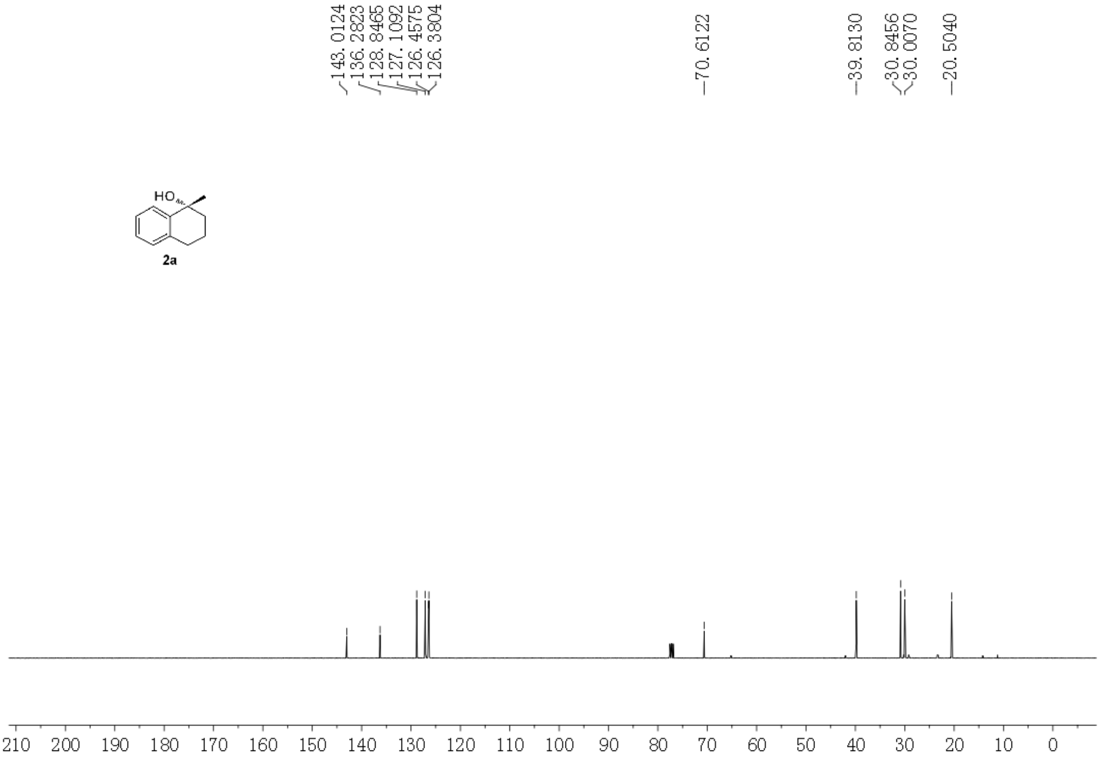

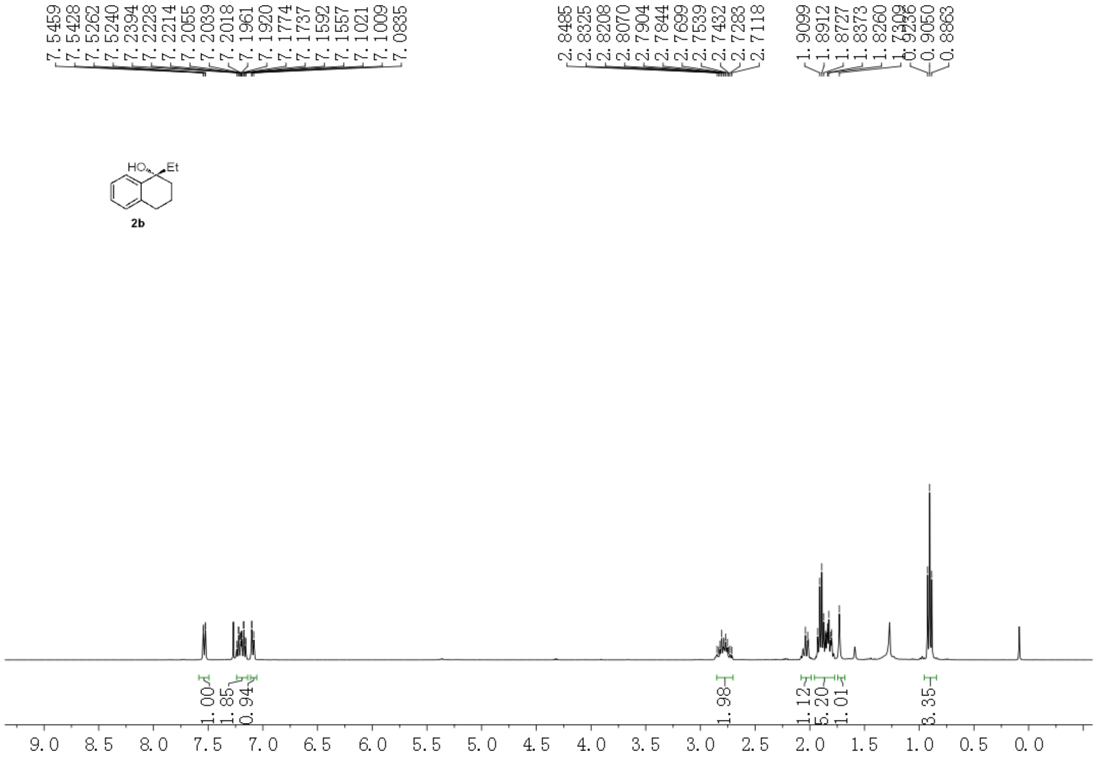
7.5459
7.5428
7.5262
7.5240
7.2394
7.2228
7.2214
7.2055
7.2039
7.2018
7.1961
7.1920
7.1774
7.1737
7.1592
7.1557
7.1021
7.1009
7.0835
2.8485
2.8325
2.8208
2.8070
2.7904
2.7844
2.7699
2.7539
2.7432
2.7283
2.7118
1.9099
1.8912
1.8727
1.8373
1.8260
0.9050
0.8863
HO
Et
2b
1.00
1.85
0.94
1.98
1.12
5.20
1.01
3.35
9.0 8.5 8.0 7.5 7.0 6.5 6.0 5.5 5.0 4.5 4.0 3.5 3.0 2.5 2.0 1.5 1.0 0.5 0.0

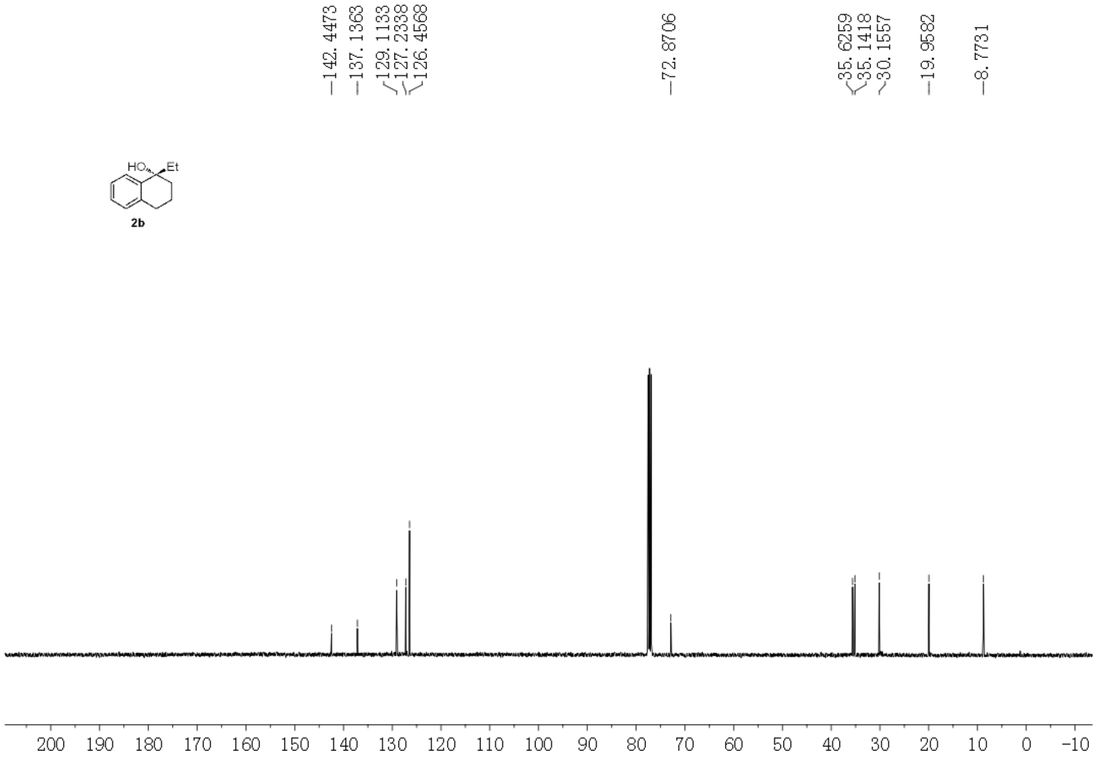
142.4473
137.1363
129.1133
127.2338
126.4568
72.8706
35.6259
35.1418
30.1557
19.9582
8.7731
HO
Et
2b
200 190 180 170 160 150 140 130 120 110 100 90 80 70 60 50 40 30 20 10 0 -10

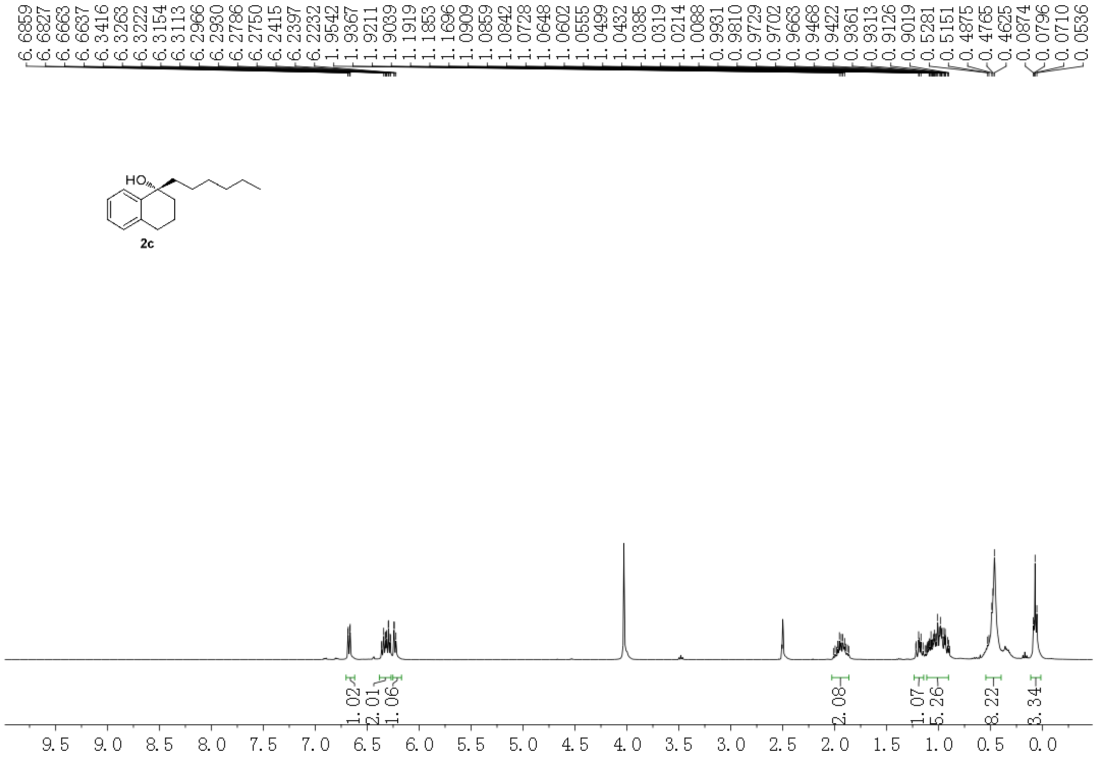
6.6859
6.6827
6.6663
6.6637
6.3416
6.3263
6.3222
6.3154
6.3113
6.2966
6.2930
6.2786
6.2750
6.2415
6.2397
6.2232
1.9542
1.9367
1.9211
1.9039
1.1919
1.1853
1.1696
1.0909
1.0859
1.0842
1.0728
1.0648
1.0602
1.0555
1.0499
1.0432
1.0385
1.0319
1.0214
1.0088
0.9931
0.9810
0.9729
0.9702
0.9663
0.9468
0.9422
0.9361
0.9313
0.9126
0.9019
0.5281
0.5151
0.4875
0.4765
0.4625
0.0874
0.0796
0.0710
0.0536
1.02
2.01
1.06
2.08
1.07
5.26
8.22
3.34
9.5 9.0 8.5 8.0 7.5 7.0 6.5 6.0 5.5 5.0 4.5 4.0 3.5 3.0 2.5 2.0 1.5 1.0 0.5 0.0
HO

2c

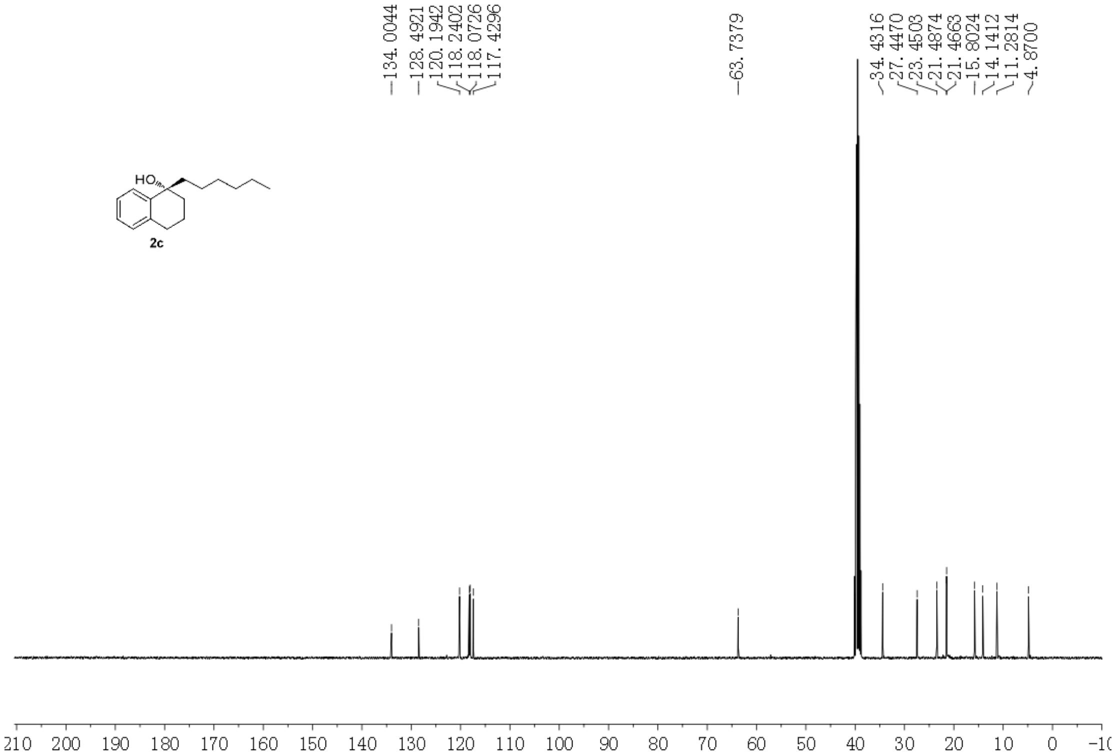
134.0044
128.4921
120.1942
118.2402
118.0726
117.4296
63.7379
34.4316
27.4470
23.4503
21.4874
21.4663
15.8024
14.1412
11.2814
4.8700
210 200 190 180 170 160 150 140 130 120 110 100 90 80 70 60 50 40 30 20 10 0 -1(
HO

2c

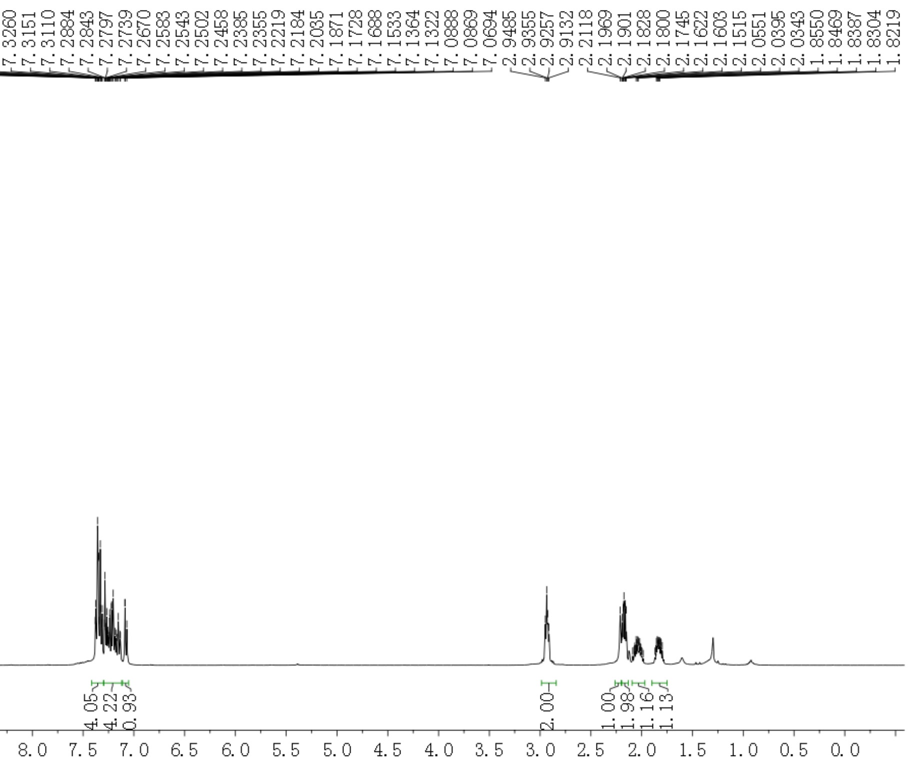
7.3800
7.3754
7.3698
7.3583
7.3548
7.3498
7.3479
7.3424
7.3305
7.3260
7.3151
7.3110
7.2884
7.2843
7.2797
7.2739
7.2670
7.2583
7.2543
7.2502
7.2458
7.2385
7.2355
7.2219
7.2184
7.2035
7.1871
7.1728
7.1688
7.1533
7.1364
7.1322
7.0888
7.0869
7.0694
2.9485
2.9355
2.9257
2.9132
2.2118
2.1969
2.1901
2.1828
2.1800
2.1745
2.1622
2.1603
2.1515
2.0551
2.0395
2.0343
1.8550
1.8469
1.8387
1.8304
1.8219
4.05
4.22
0.93
2.00
1.00
1.98
1.16
1.13
9.5 9.0 8.5 8.0 7.5 7.0 6.5 6.0 5.5 5.0 4.5 4.0 3.5 3.0 2.5 2.0 1.5 1.0 0.5 0.0
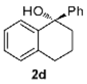
HO
Ph

2d

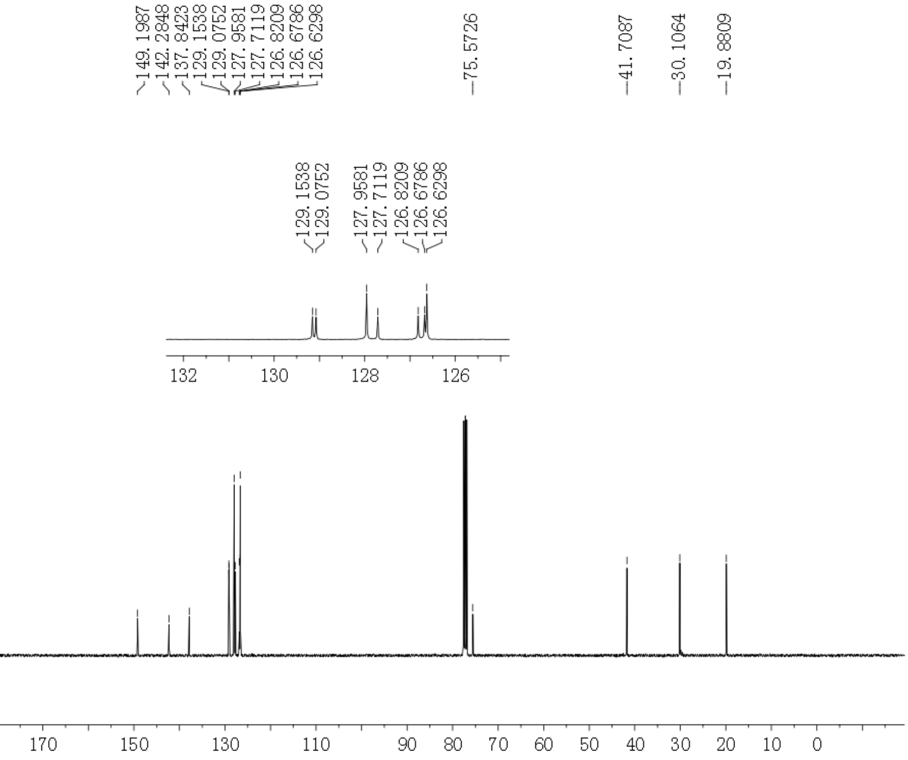
149.1987
142.2848
137.8423
129.1538
129.0752
127.9581
127.7119
126.8209
126.6786
126.6298
75.5726
41.7087
30.1064
19.8809
129.1538
129.0752
127.9581
127.7119
126.8209
126.6786
126.6298
132 130 128 126
210 190 170 150 130 110 90 80 70 60 50 40 30 20 10 0
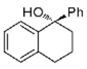
HO
Ph

2d

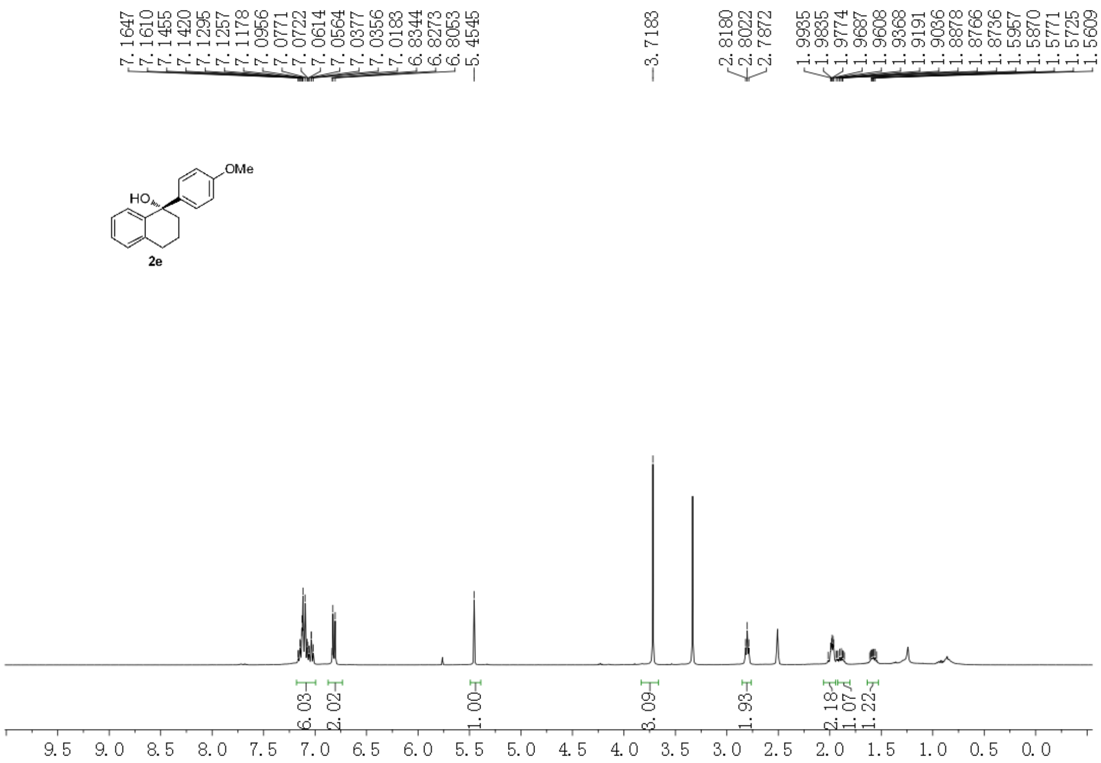

7.1647
7.1610
7.1455
7.1420
7.1295
7.1257
7.1178
7.0956
7.0771
7.0722
7.0614
7.0564
7.0377
7.0356
7.0183
6.8344
6.8273
6.8053
5.4545
3.7183
2.8180
2.8022
2.7872
1.9935
1.9835
1.9774
1.9687
1.9608
1.9368
1.9191
1.9036
1.8878
1.8766
1.8736
1.5957
1.5870
1.5771
1.5725
1.5609
OMe
HO
2e
6.03
2.02
1.00
3.09
1.93
2.18
1.07
1.22
9.5 9.0 8.5 8.0 7.5 7.0 6.5 6.0 5.5 5.0 4.5 4.0 3.5 3.0 2.5 2.0 1.5 1.0 0.5 0.0

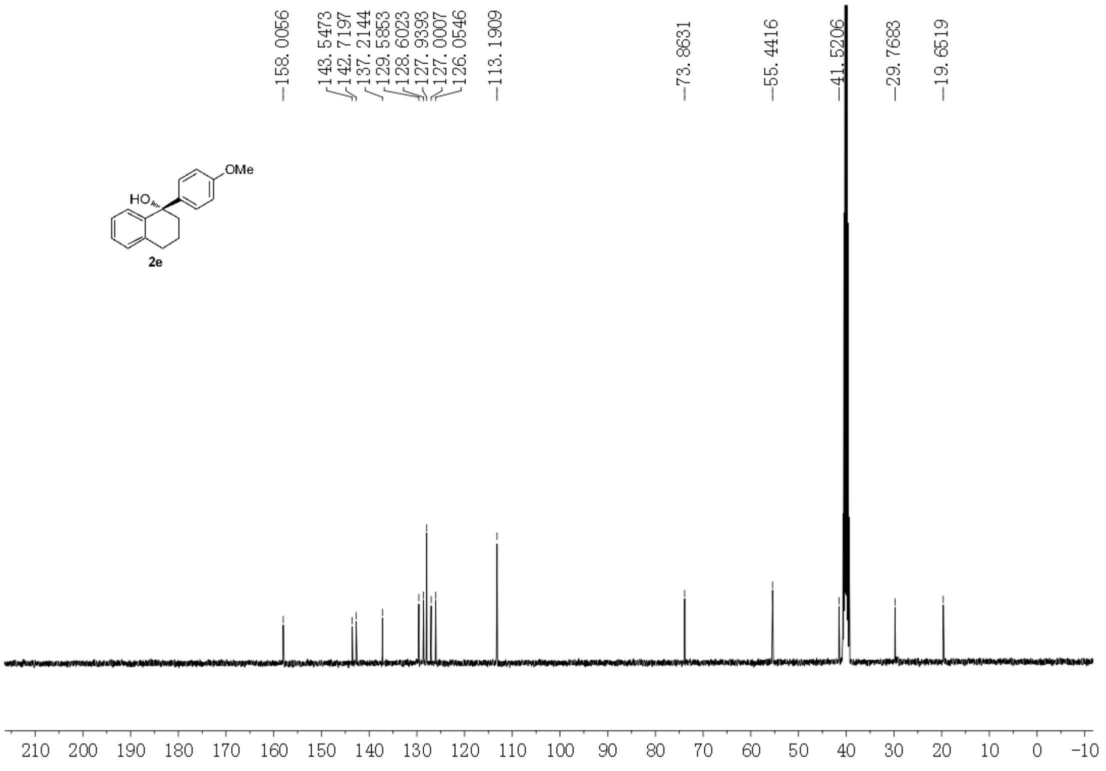

158.0056
143.5473
142.7197
137.2144
129.5853
128.6023
127.9393
127.0007
126.0546
113.1909
73.8631
55.4416
41.5206
29.7683
19.6519
OMe
HO
2e
210 200 190 180 170 160 150 140 130 120 110 100 90 80 70 60 50 40 30 20 10 0 -10

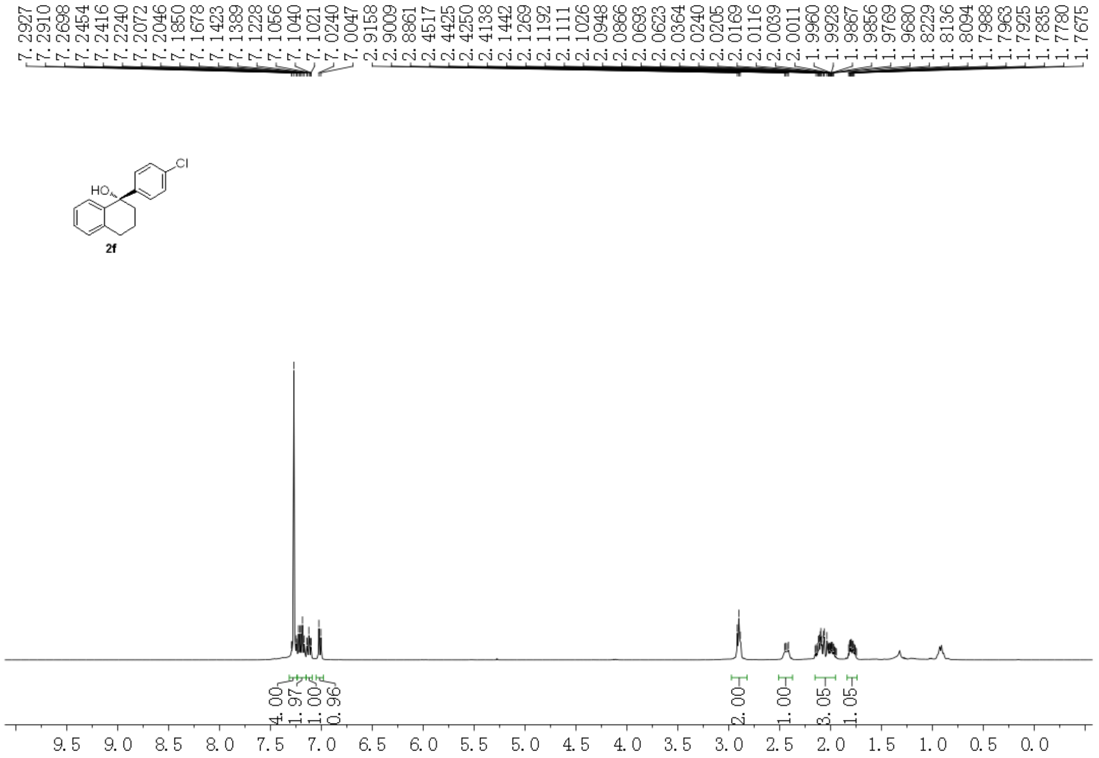
7.2927
7.2910
7.2698
7.2454
7.2416
7.2240
7.2072
7.2046
7.1850
7.1678
7.1423
7.1389
7.1228
7.1056
7.1040
7.1021
7.0240
7.0047
2.9158
2.9009
2.8861
2.4517
2.4425
2.4250
2.4138
2.1442
2.1269
2.1192
2.1111
2.1026
2.0948
2.0866
2.0693
2.0623
2.0364
2.0240
2.0205
2.0169
2.0116
2.0039
2.0011
1.9960
1.9928
1.9867
1.9856
1.9769
1.9680
1.8229
1.8136
1.8094
1.7988
1.7963
1.7925
1.7835
1.7780
1.7675
HO
Cl
2f
4.00
1.97
1.00
0.96
2.00
1.00
3.05
1.05
9.5 9.0 8.5 8.0 7.5 7.0 6.5 6.0 5.5 5.0 4.5 4.0 3.5 3.0 2.5 2.0 1.5 1.0 0.5 0.0

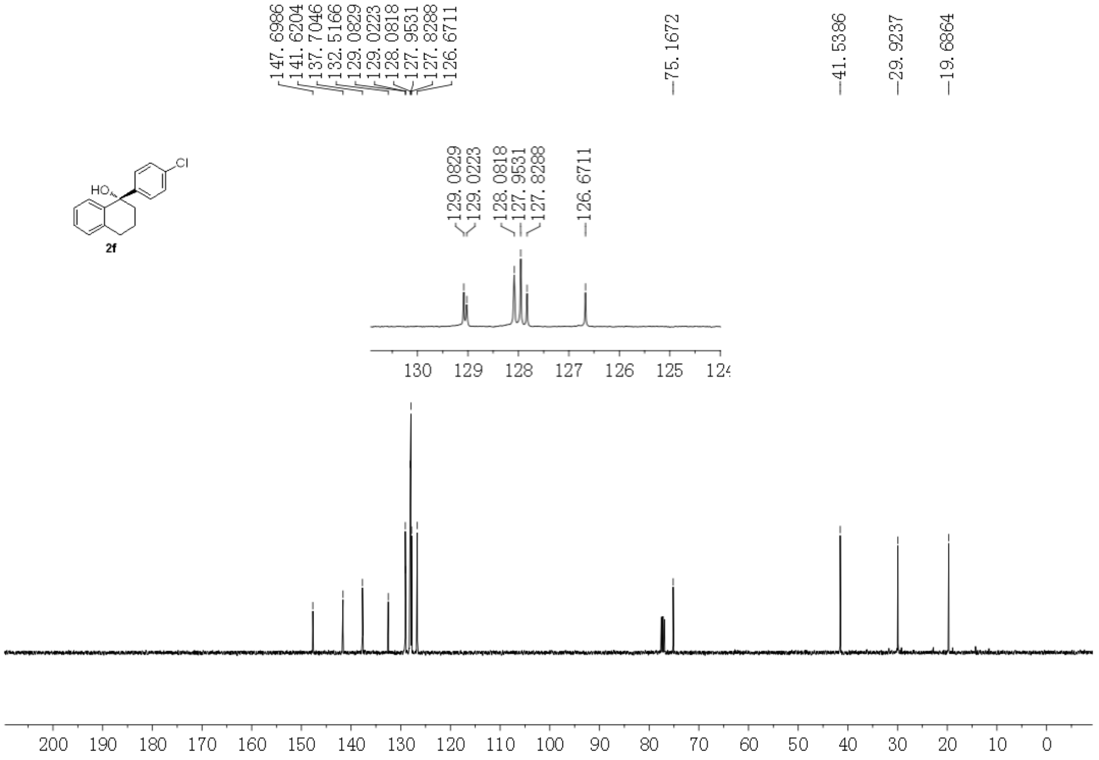
147.6986
141.6204
137.7046
132.5166
129.0829
129.0223
128.0818
127.9531
127.8288
126.6711
75.1672
41.5386
29.9237
19.6864
HO
Cl
2f
129.0829
129.0223
128.0818
127.9531
127.8288
126.6711
130 129 128 127 126 125 12
200 190 180 170 160 150 140 130 120 110 100 90 80 70 60 50 40 30 20 10 0

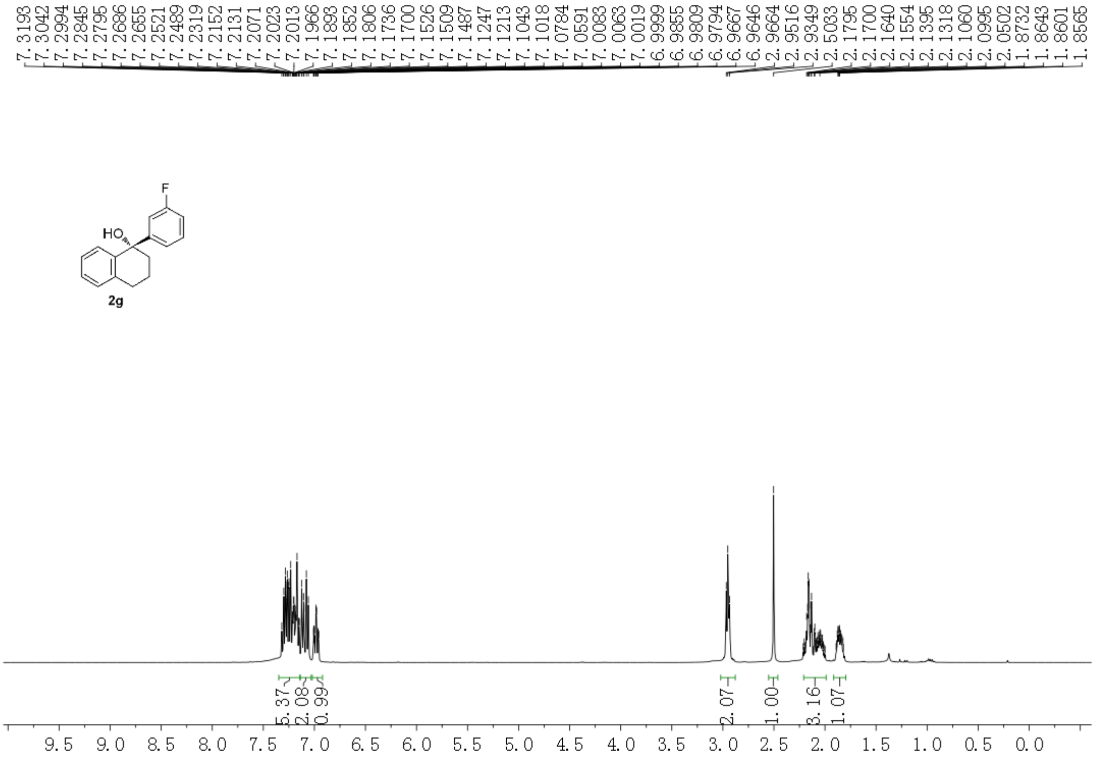
7.3193 7.3042 7.2994 7.2845 7.2795 7.2686 7.2655 7.2521 7.2489 7.2319 7.2152 7.2131 7.2071 7.2023 7.2013 7.1966 7.1893 7.1852 7.1806 7.1736 7.1700 7.1526 7.1509 7.1487 7.1247 7.1213 7.1043 7.1018 7.0784 7.0591 7.0083 7.0063 7.0019 6.9999 6.9855 6.9809 6.9794 6.9667 6.9646 2.9664 2.9516 2.9349 2.5033 2.1795 2.1700 2.1640 2.1554 2.1395 2.1318 2.1060 2.0995 2.0502 1.8732 1.8643 1.8601 1.8565
F
HO
2g
5.37
2.08
0.99
2.07
1.00
3.16
1.07
9.5 9.0 8.5 8.0 7.5 7.0 6.5 6.0 5.5 5.0 4.5 4.0 3.5 3.0 2.5 2.0 1.5 1.0 0.5 0.0

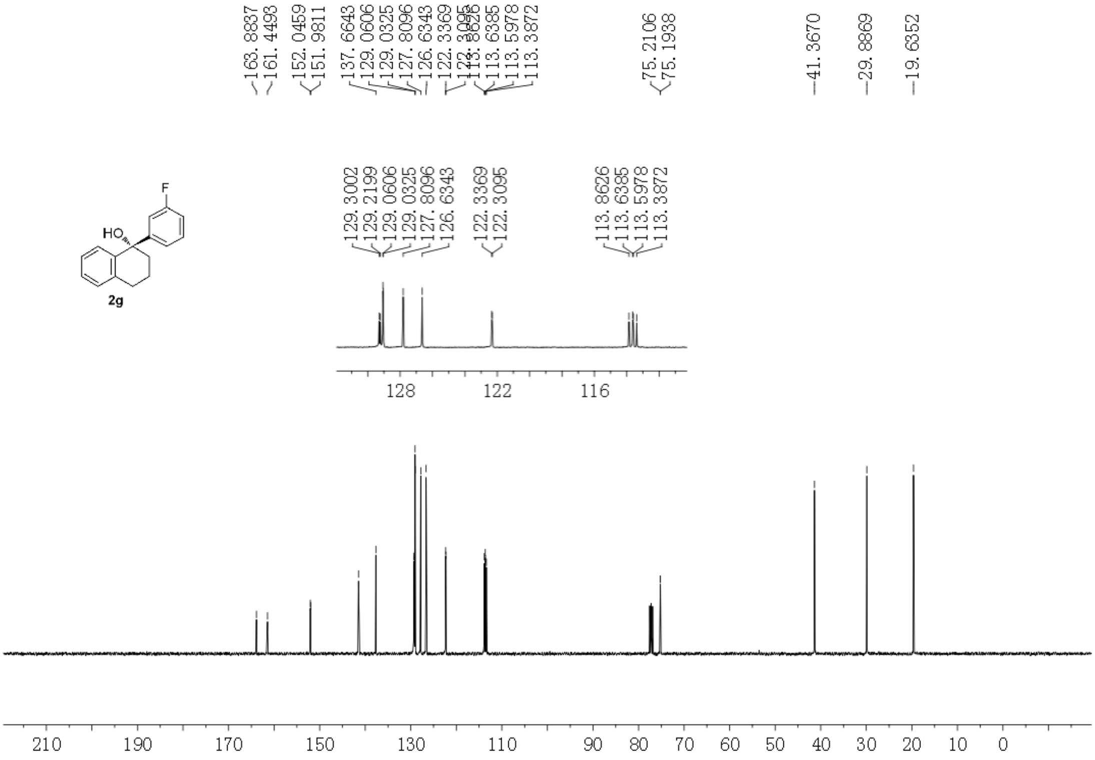
163.8837 161.4493 152.0459 151.9811 137.6643 129.0606 129.0325 127.8096 126.6343 122.3369 122.3095 113.8626 113.6385 113.5978 113.3872 75.2106 75.1938 41.3670 29.8869 19.6352
F
HO
2g
129.3002 129.2199 129.0606 129.0325 127.8096 126.6343 122.3369 122.3095 113.8626 113.6385 113.5978 113.3872
128 122 116
210 190 170 150 130 110 90 80 70 60 50 40 30 20 10 0

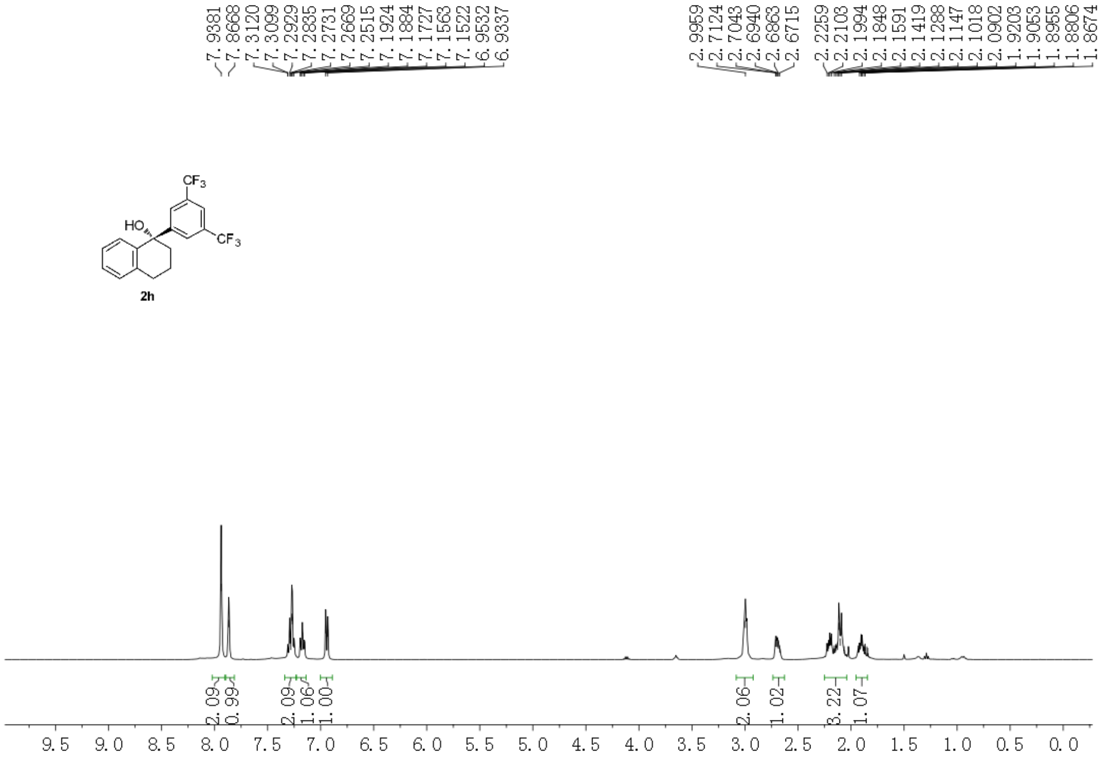
7.9381
7.8668
7.3120
7.3099
7.2929
7.2835
7.2731
7.2669
7.2515
7.1924
7.1884
7.1727
7.1563
7.1522
6.9532
6.9337
2.9959
2.7124
2.7043
2.6940
2.6863
2.6715
2.2259
2.2103
2.1994
2.1848
2.1591
2.1419
2.1288
2.1147
2.1018
2.0902
1.9203
1.9053
1.8955
1.8806
1.8674
2.09
0.99
2.09
1.06
1.00
2.06
1.02
3.22
1.07
9.5 9.0 8.5 8.0 7.5 7.0 6.5 6.0 5.5 5.0 4.5 4.0 3.5 3.0 2.5 2.0 1.5 1.0 0.5 0.0
CF3
HO
CF3

2h

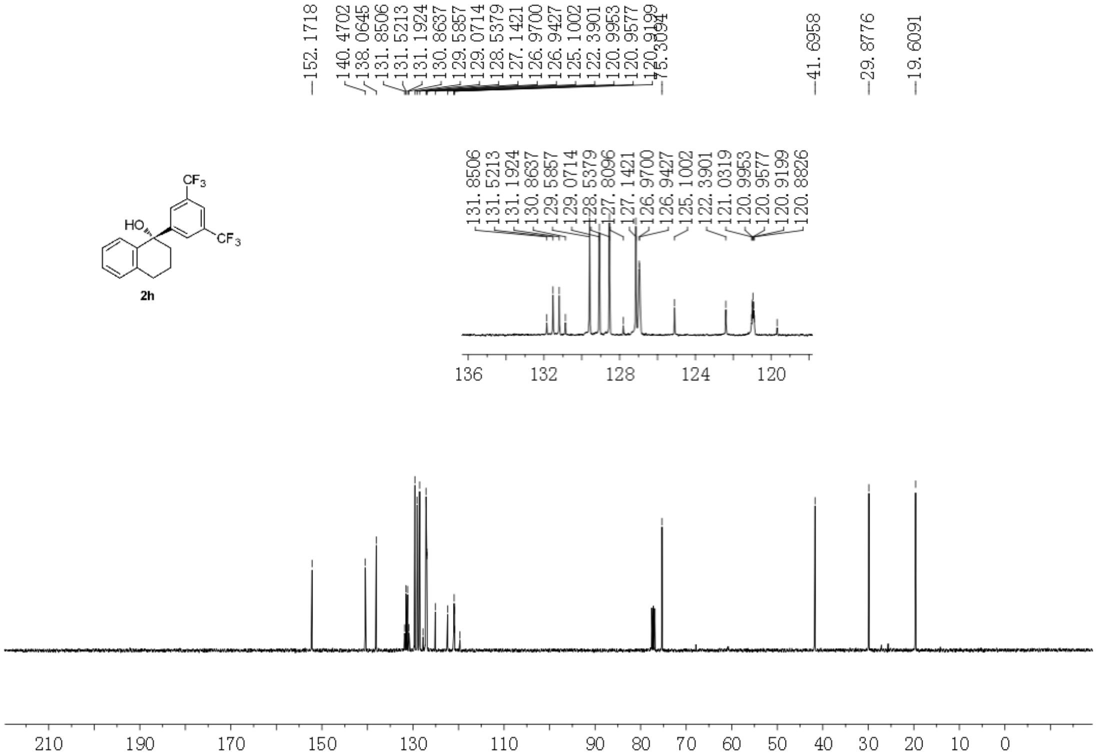
152.1718
140.4702
138.0645
131.8506
131.5213
131.1924
130.8637
129.5857
129.0714
128.5379
127.1421
126.9700
126.9427
125.1002
122.3901
120.9953
120.9577
120.9199
75.3094
41.6958
29.8776
19.6091
131.8506
131.5213
131.1924
130.8637
129.5857
128.5379
129.0714
127.8096
127.1421
126.9700
126.9427
125.1002
122.3901
121.0319
120.9953
120.9577
120.9199
120.8826
136 132 128 124 120
210 190 170 150 130 110 90 80 70 60 50 40 30 20 10 0
CF3
HO
CF3

2h

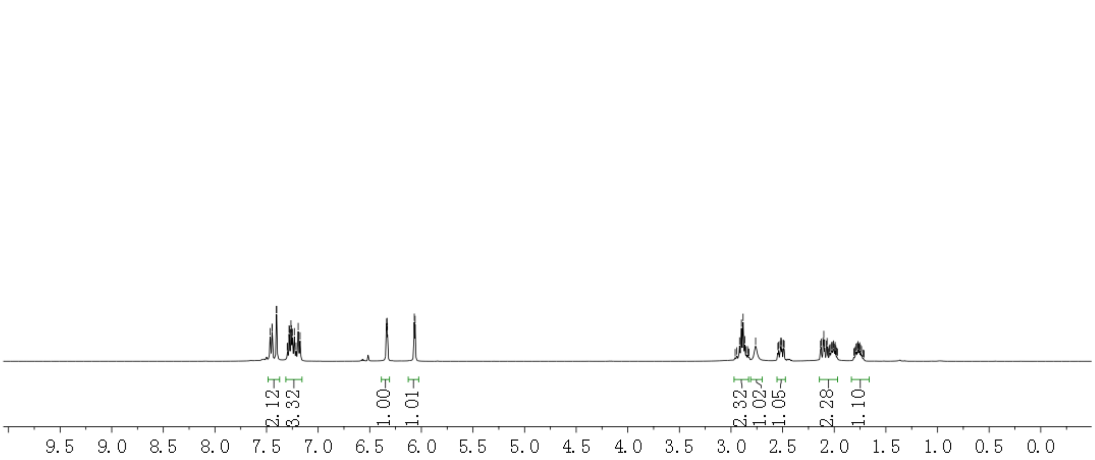
7.4657
7.4473
7.4449
7.4040
7.4023
7.2996
7.2964
7.2819
7.2781
7.2637
7.2599
7.2484
7.2301
7.2144
7.1933
7.1748
6.3410
6.3364
6.3333
6.3288
6.0684
6.0607
2.9174
2.8991
2.8831
2.8672
2.7620
2.5462
2.5389
2.5248
2.5169
2.5143
2.5063
2.4921
2.4852
2.1329
2.1258
2.1091
2.1014
2.0932
2.0765
2.0693
2.0405
2.0252
2.0212
2.0123
2.0068
1.9995
1.9912
1.7880
1.7773
1.7695
1.7649
1.7616
1.7538
1.7458
2.12
3.32
1.00
1.01
2.32
1.02
1.05
2.28
1.10
9.5 9.0 8.5 8.0 7.5 7.0 6.5 6.0 5.5 5.0 4.5 4.0 3.5 3.0 2.5 2.0 1.5 1.0 0.5 0.0

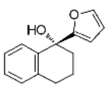

2i

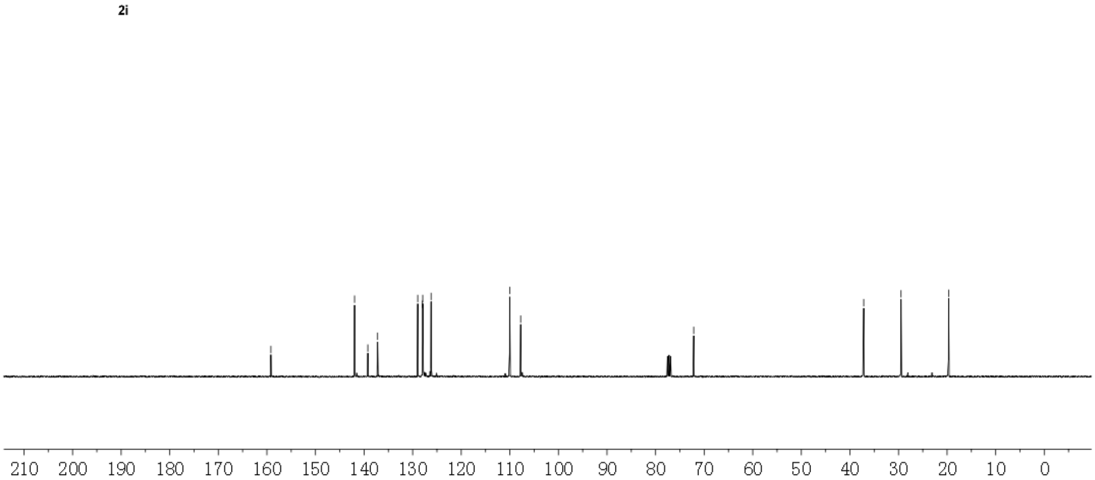
159.1818
141.9599
139.2275
137.2170
128.9541
127.9923
127.9223
126.2045
110.0315
107.7670
72.1501
37.1576
29.4778
19.6826
210 200 190 180 170 160 150 140 130 120 110 100 90 80 70 60 50 40 30 20 10 0

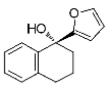

2i

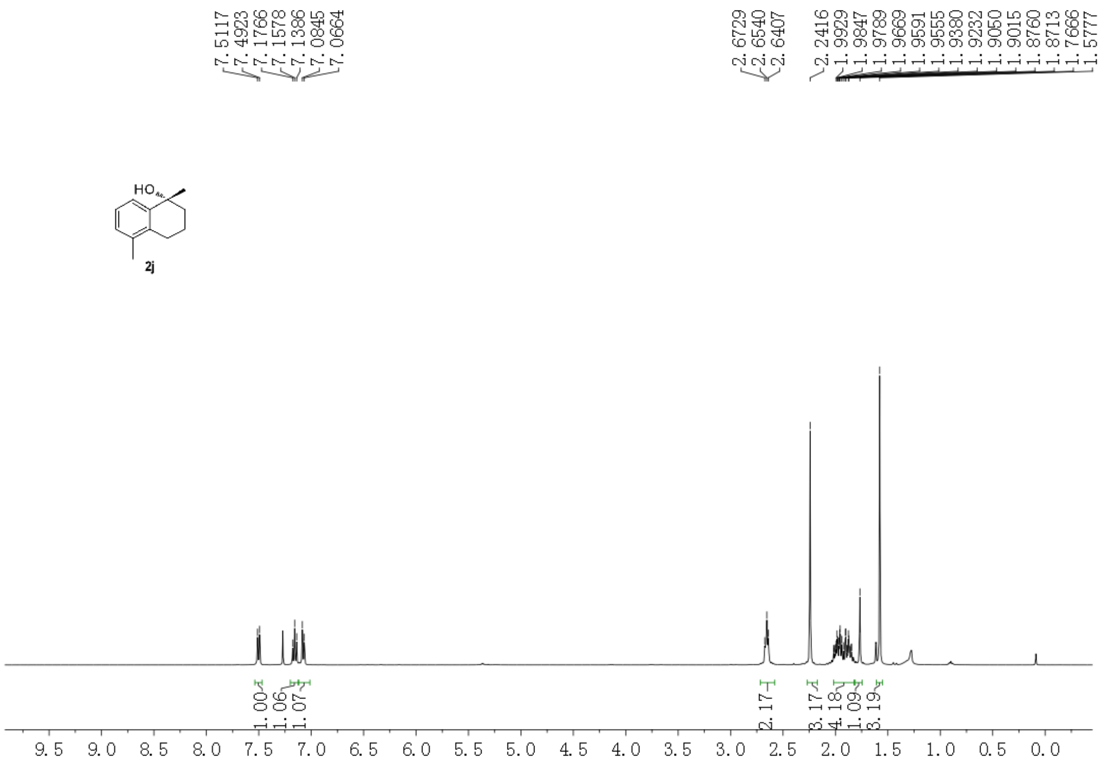
7.5117
7.4923
7.1766
7.1578
7.1386
7.0845
7.0664
2.6729
2.6540
2.6407
2.2416
1.9929
1.9847
1.9789
1.9669
1.9591
1.9555
1.9380
1.9232
1.9050
1.9015
1.8760
1.8713
1.7666
1.5777
HO
2j
1.00
1.06
1.07
2.17
3.17
4.18
1.09
3.19

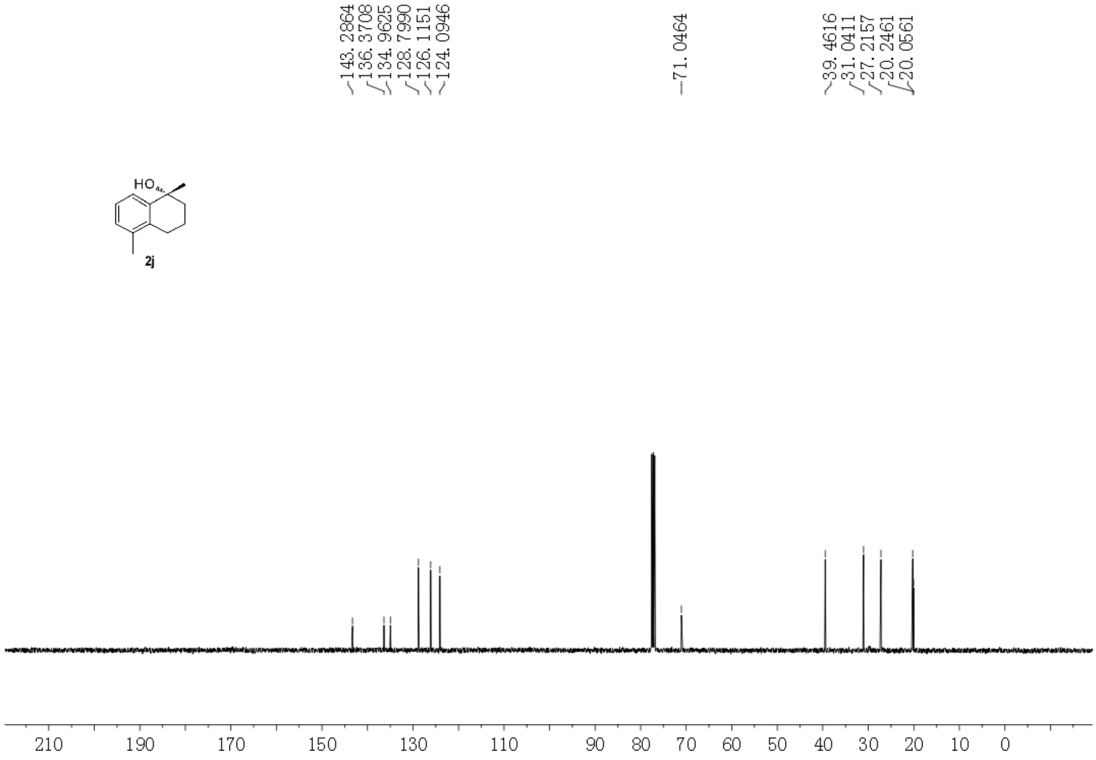
143.2864
136.3708
134.9625
128.7990
126.1151
124.0946
71.0464
39.4616
31.0411
27.2157
20.2461
20.0561
HO
2j

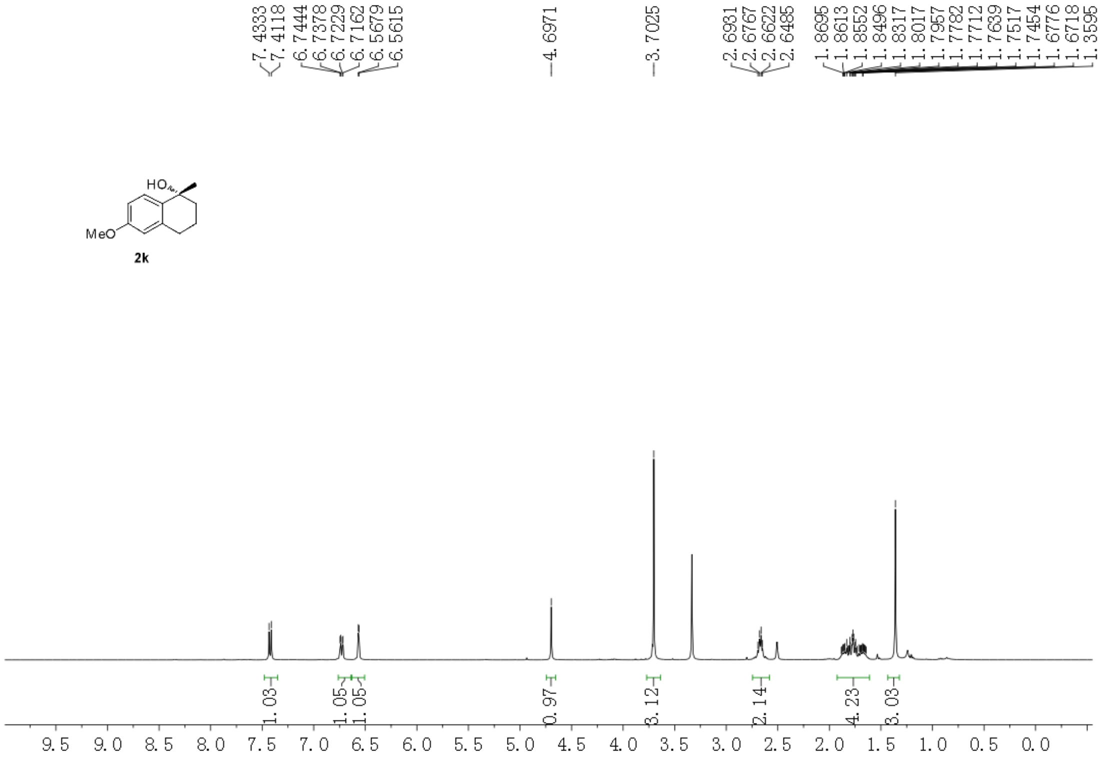
7.4333
7.4118
6.7444
6.7378
6.7229
6.7162
6.5679
6.5615
4.6971
3.7025
2.6931
2.6767
2.6622
2.6485
1.8695
1.8613
1.8552
1.8496
1.8317
1.8017
1.7957
1.7782
1.7712
1.7639
1.7517
1.7454
1.6776
1.6718
1.3595
HO
MeO
2k
1.03
1.05
1.05
0.97
3.12
2.14
4.23
3.03
9.5 9.0 8.5 8.0 7.5 7.0 6.5 6.0 5.5 5.0 4.5 4.0 3.5 3.0 2.5 2.0 1.5 1.0 0.5 0.0

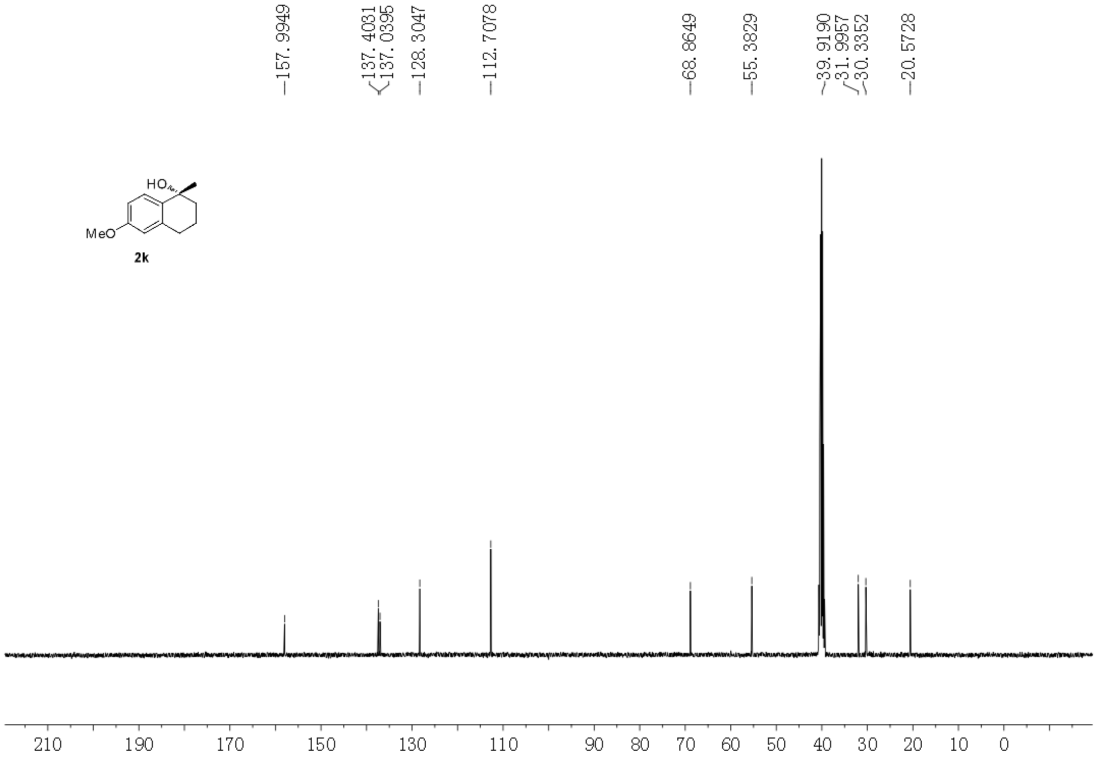
157.9949
137.4031
137.0395
128.3047
112.7078
68.8649
55.3829
39.9190
31.9957
30.3352
20.5728
HO
MeO
2k
210 190 170 150 130 110 90 80 70 60 50 40 30 20 10 0

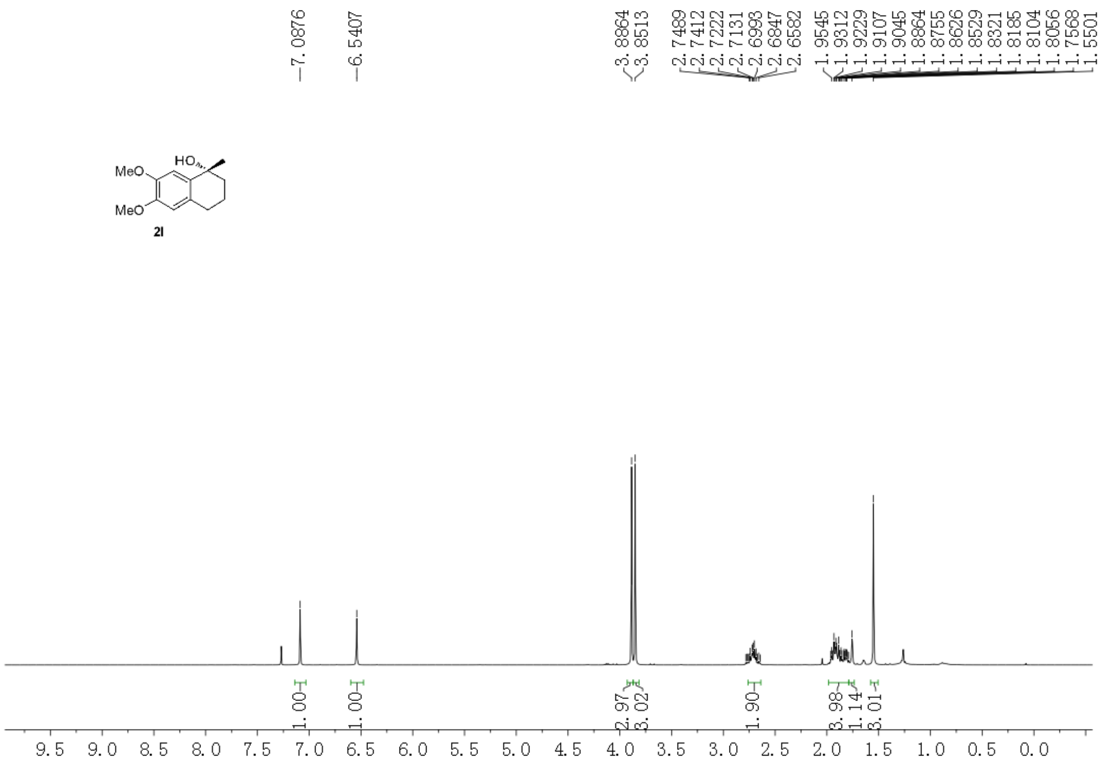
7.0876
6.5407
3.8864
3.8513
2.7489
2.7412
2.7222
2.7131
2.6993
2.6847
2.6582
1.9545
1.9312
1.9229
1.9107
1.9045
1.8864
1.8755
1.8626
1.8529
1.8321
1.8185
1.8104
1.8056
1.7568
1.5501
MeO
MeO
HO
2l
1.00
1.00
2.97
3.02
1.90
3.98
1.14
3.01
9.5 9.0 8.5 8.0 7.5 7.0 6.5 6.0 5.5 5.0 4.5 4.0 3.5 3.0 2.5 2.0 1.5 1.0 0.5 0.0

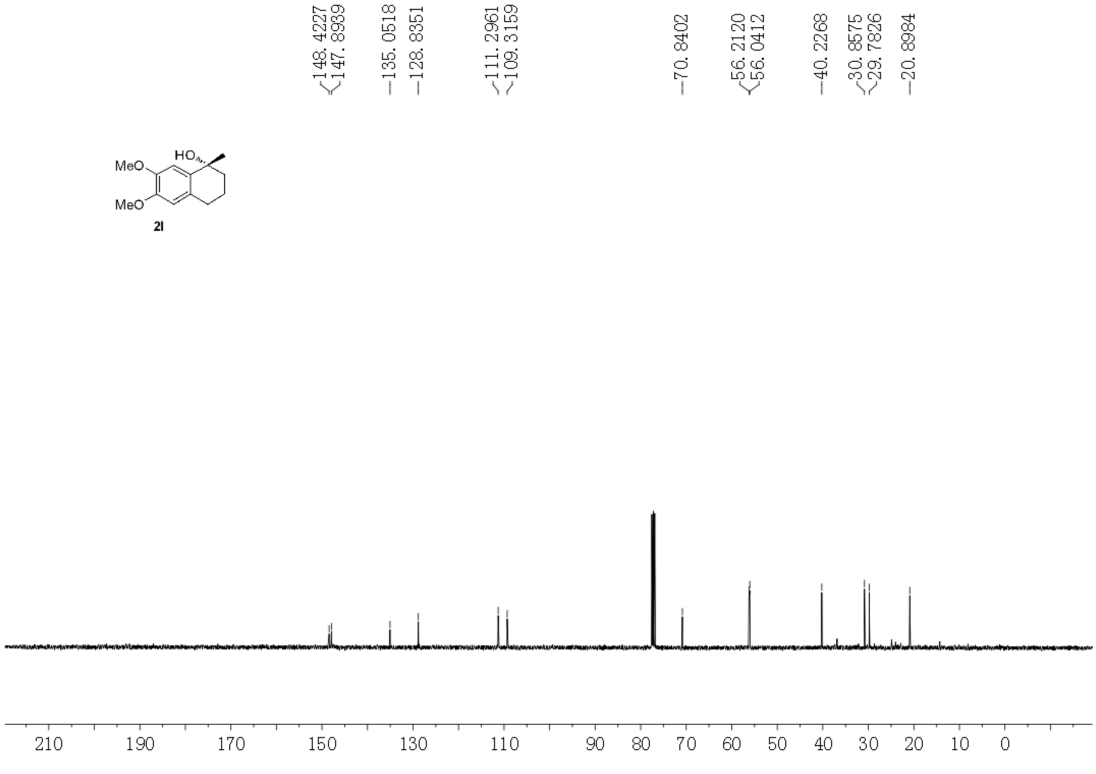
148.4227
147.8939
135.0518
128.8351
111.2961
109.3159
70.8402
56.2120
56.0412
40.2268
30.8575
29.7826
20.8984
MeO
MeO
HO
2l
210 190 170 150 130 110 90 80 70 60 50 40 30 20 10 0

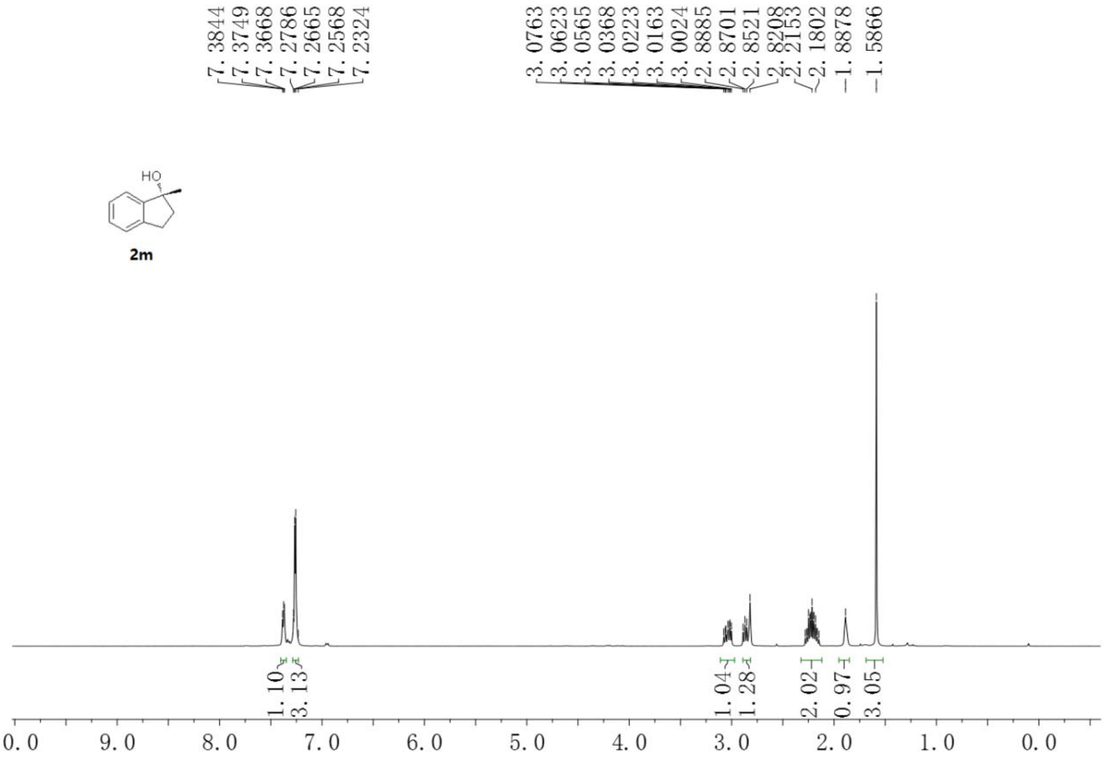
7.3844
7.3749
7.3668
7.2786
7.2665
7.2568
7.2324
3.0763
3.0623
3.0565
3.0368
3.0223
3.0163
3.0024
2.8885
2.8701
2.8521
2.8208
2.2153
2.1802
1.8878
1.5866
HO
2m
1.10
3.13
1.04
1.28
2.02
0.97
3.05
0.0
9.0
8.0
7.0
6.0
5.0
4.0
3.0
2.0
1.0
0.0

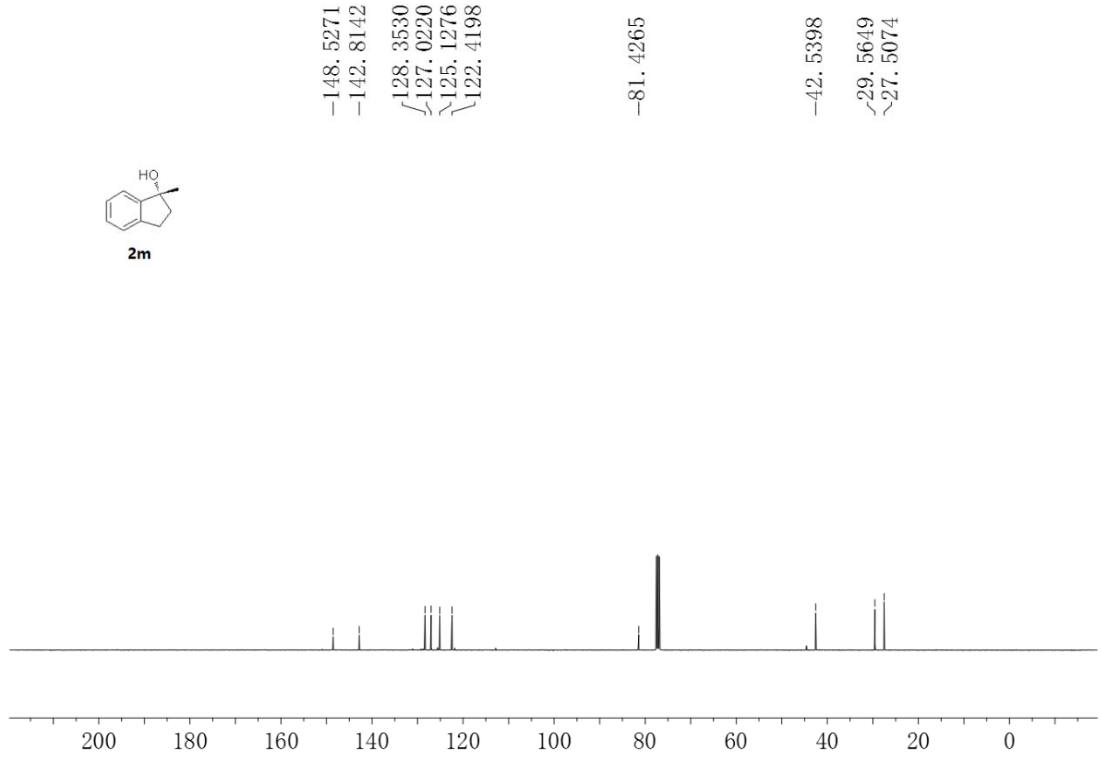
148.5271
142.8142
128.3530
127.0220
125.1276
122.4198
81.4265
42.5398
29.5649
27.5074
HO
2m
200
180
160
140
120
100
80
60
40
20
0

HPLC data

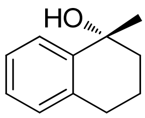

(*R*)-1-Methyl-1,2,3,4-tetrahydronaphthalen-1-ol (2a)

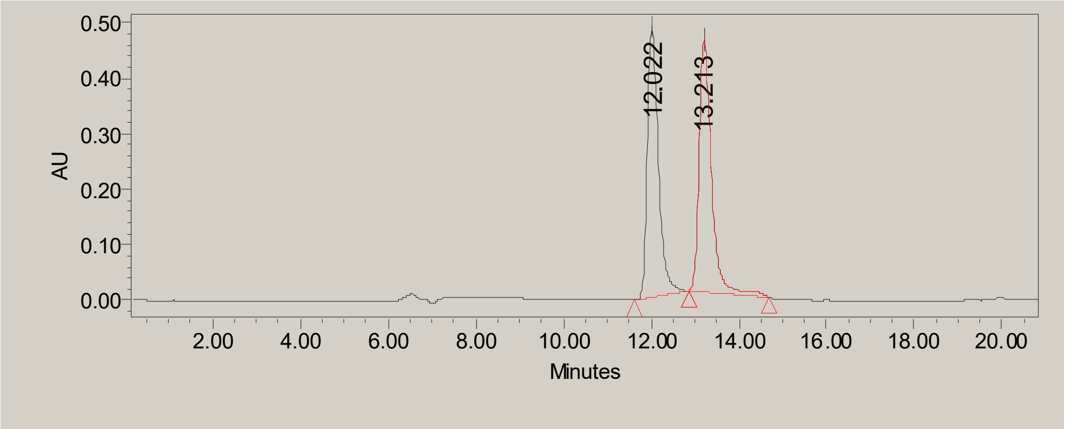

	Name	Retention Time	Area	% Area	Height	Int Type	Peak Type
1		12.022	8634674	48.89	487825	bb	Unknown
2		13.213	9027463	51.11	454563	bb	Unknown

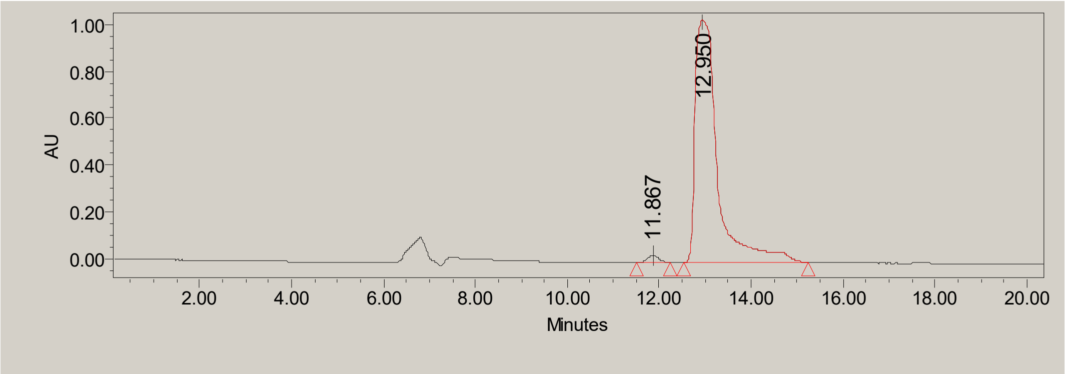

	Name	Retention Time	Area	% Area	Height	Int Type	Peak Type
1		11.867	543992	1.52	32782	bb	Unknown
2		12.950	35253642	98.48	1034932	bb	Unknown

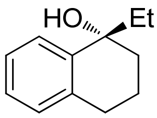

(*R*)-1-Ethyl-1,2,3,4-tetrahydronaphthalen-1-ol (2b)

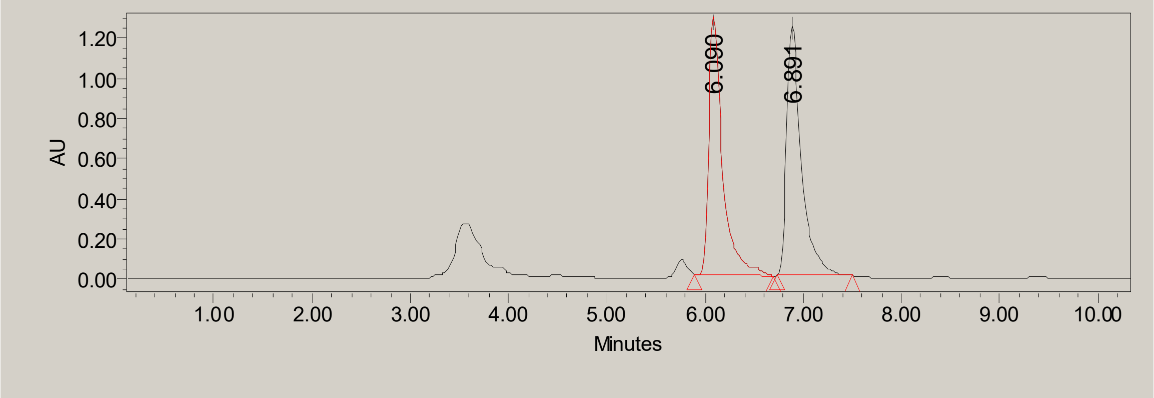

	Name	Retention Time	Area	% Area	Height	Int Type	Peak Type
1		6.090	12774860	49.44	1287205	bb	Unknown
2		6.891	13065081	50.56	1244089	bb	Unknown

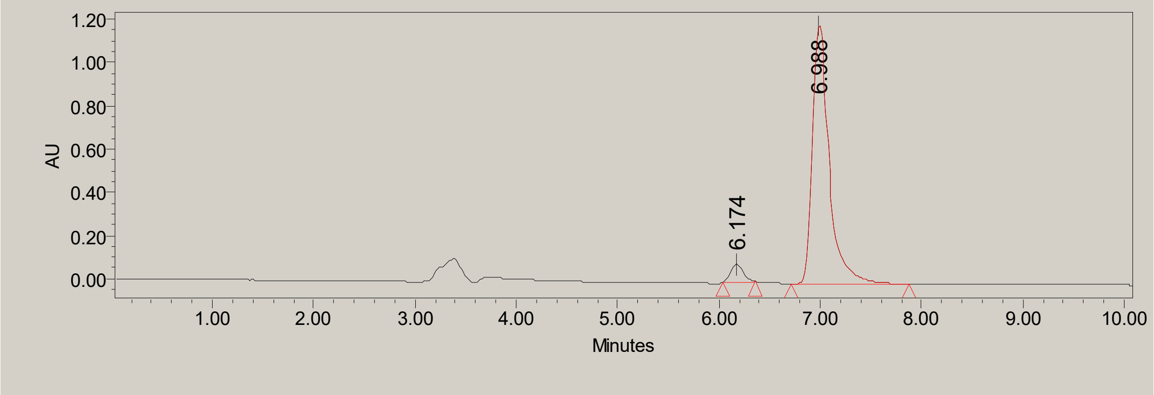

	Name	Retention Time	Area	% Area	Height	Int Type	Peak Type
1		6.174	663459	4.42	78689	bb	Unknown
2		6.988	14334693	95.58	1194927	bb	Unknown

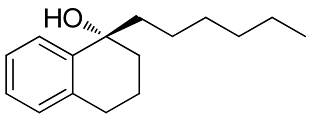

(*R*)-1-Hexyl-1,2,3,4-tetrahydronaphthalen-1-ol (2c)

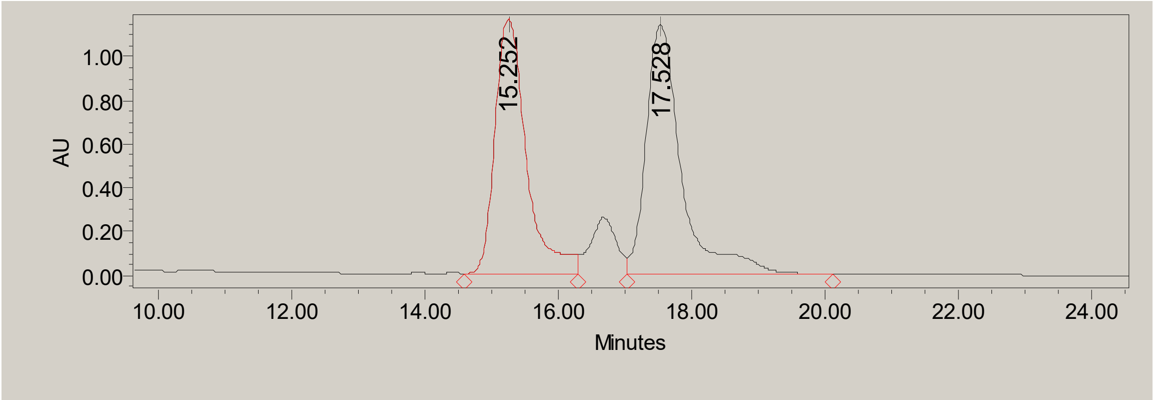

	Name	Retention Time	Area	% Area	Height	Int Type	Peak Type
1		15.252	37442760	48.26	1164378	Vv	Unknown
2		17.528	40149503	51.74	1140411	vV	Unknown

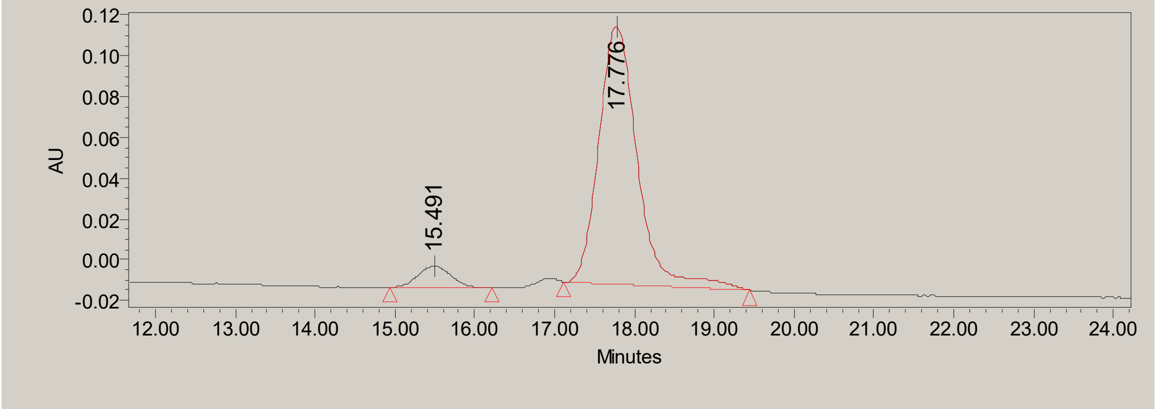

	Name	Retention Time	Area	% Area	Height	Int Type	Peak Type
1		15.491	302328	6.74	10361	bb	Unknown
2		17.776	4185587	93.26	126314	bb	Unknown

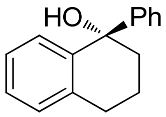

(*S*)-1-Phenyl-1,2,3,4-tetrahydronaphthalen-1-ol (2d)

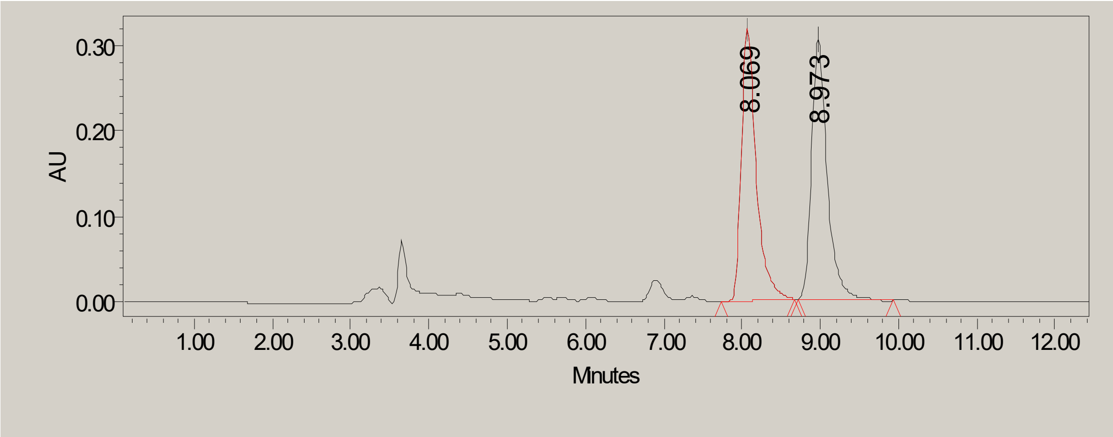

	Name	Retention Time	Area	% Area	Height	Int Type	Peak Type
1		8.069	4197906	50.01	318813	bb	Unknown
2		8.973	4195467	49.99	303051	bb	Unknown

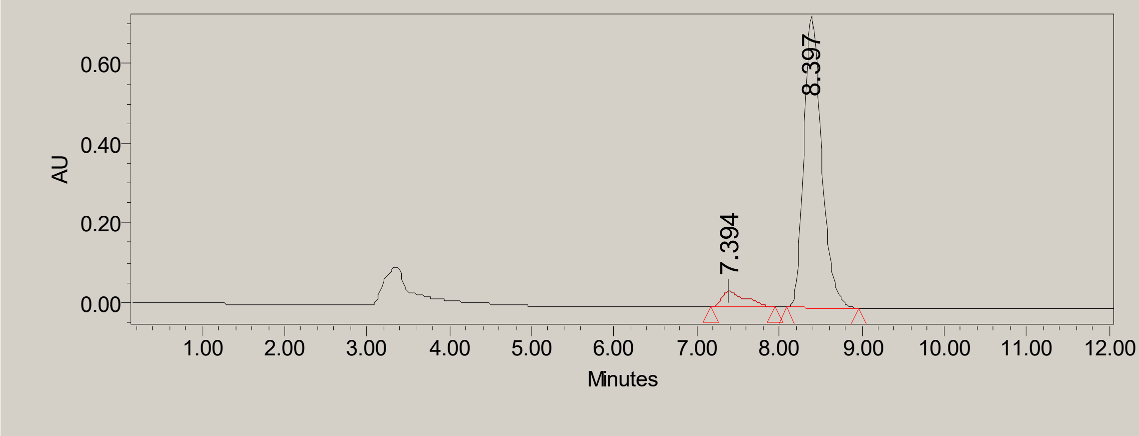

	Name	Retention Time	Area	% Area	Height	Int Type	Peak Type
1		7.394	693978	5.88	36609	bb	Unknown
2		8.397	11112312	94.12	732600	bb	Unknown

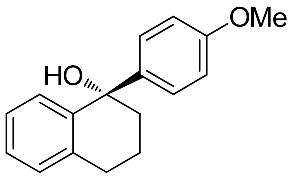

(*S*)-1-(4-Methoxyphenyl)-1,2,3,4-tetrahydronaphthalen-1-ol (2e)

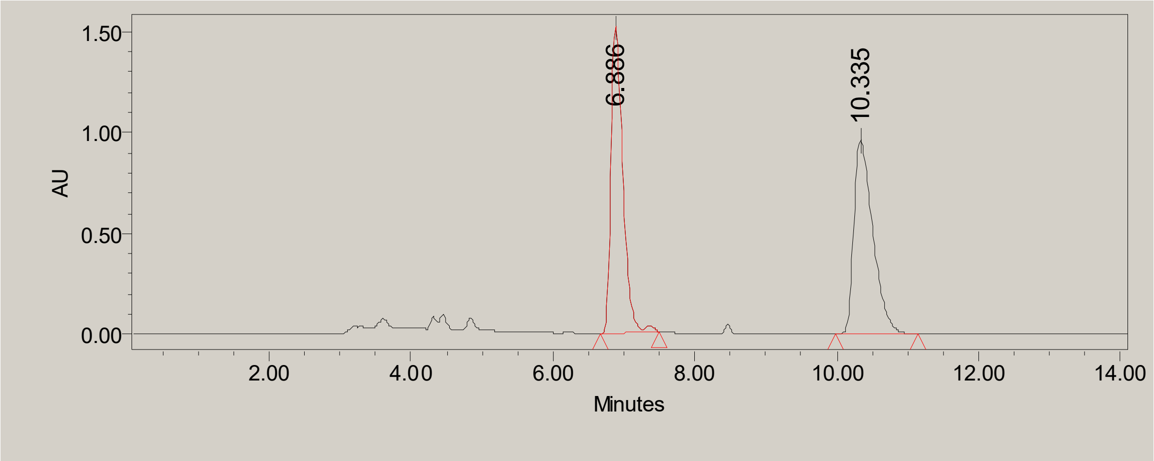

	Name	Retention Time	Area	% Area	Height	Int Type	Peak Type
1		6.886	17920580	49.82	1526496	bb	Unknown
2		10.335	18048369	50.18	960718	bb	Unknown

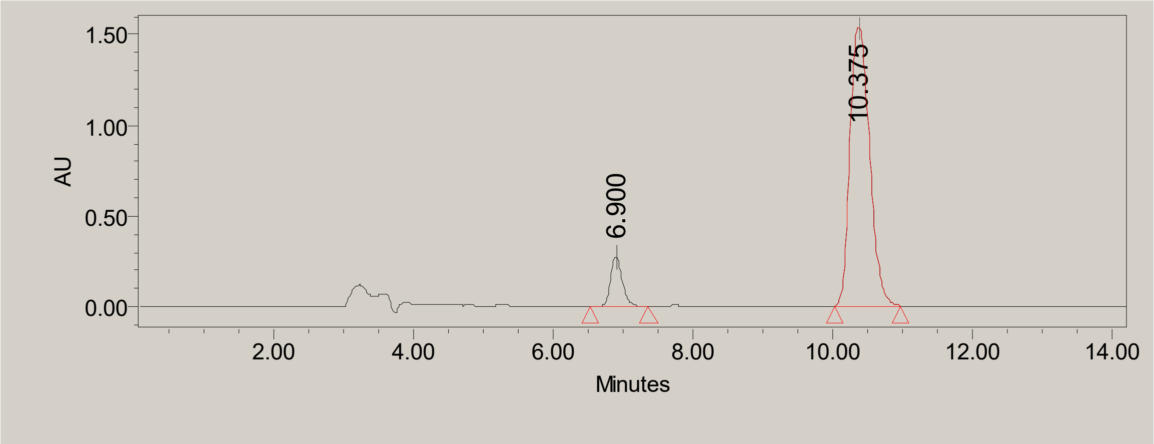

	Name	Retention Time	Area	% Area	Height	Int Type	Peak Type
1		6.900	3215708	10.98	275885	bb	Unknown
2		10.375	26070472	89.02	1428651	bb	Unknown

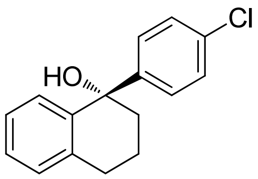

(*S*)-1-(4-Chlorophenyl)-1,2,3,4-tetrahydronaphthalen-1-ol (2f)

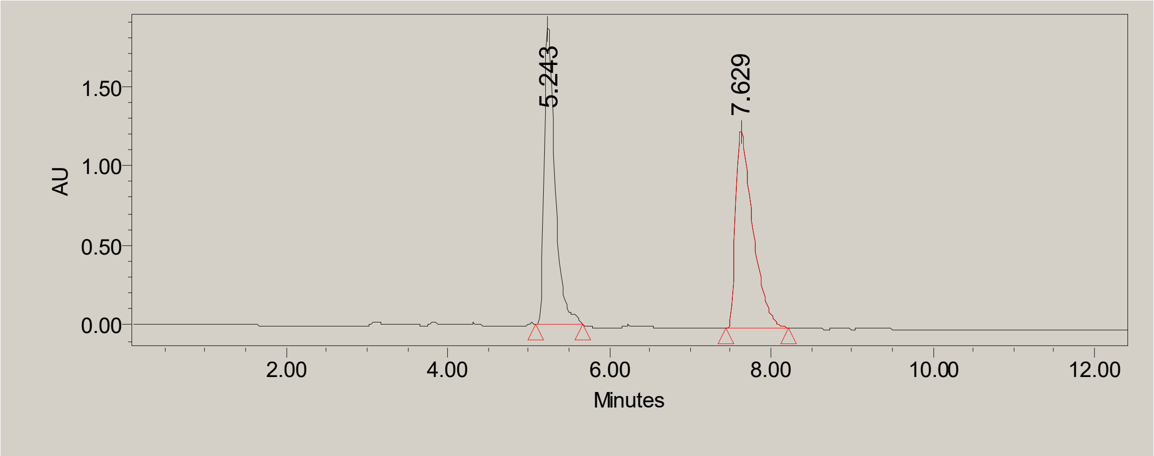

	Name	Retention Time	Area	% Area	Height	Int Type	Peak Type
1		5.243	18558584	50.21	1898906	bb	Unknown
2		7.629	18405718	49.79	1230536	bb	Unknown

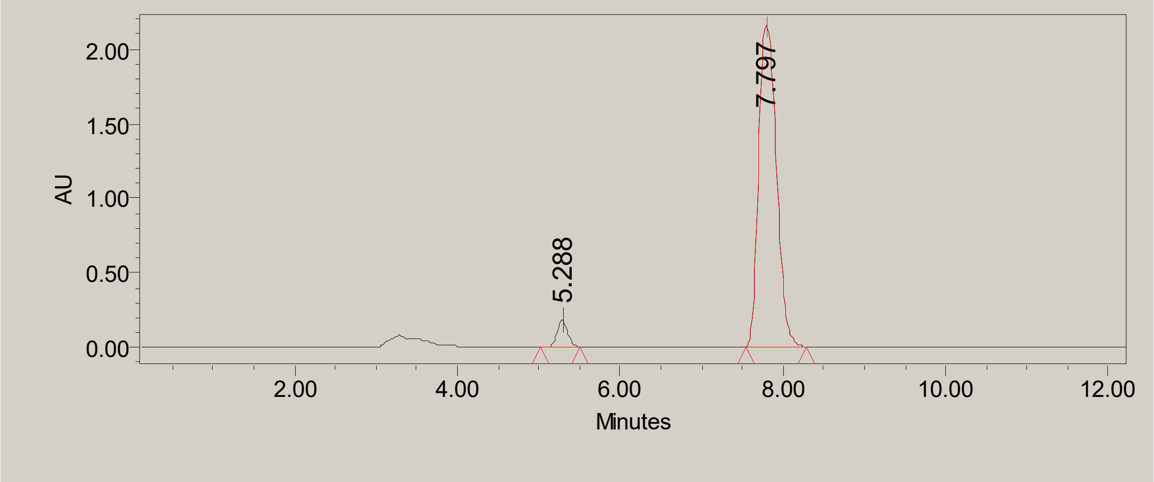

	Name	Retention Time	Area	% Area	Height	Int Type	Peak Type
1		5.288	1608034	4.73	178159	bb	Unknown
2		7.797	32411250	95.27	2162109	bb	Unknown

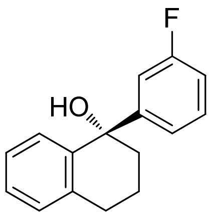

(*S*)-1-(3-Fluorophenyl)-1,2,3,4-tetrahydronaphthalen-1-ol (2g)

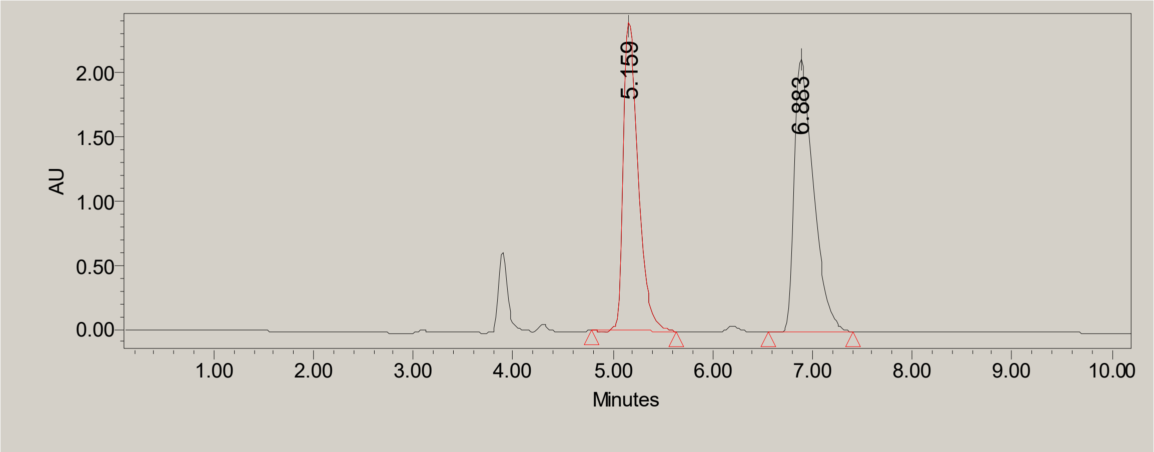

	Name	Retention Time	Area	% Area	Height	Int Type	Peak Type
1		5.159	25063723	48.66	2417245	bb	Unknown
2		6.883	26439387	51.34	2071720	bb	Unknown

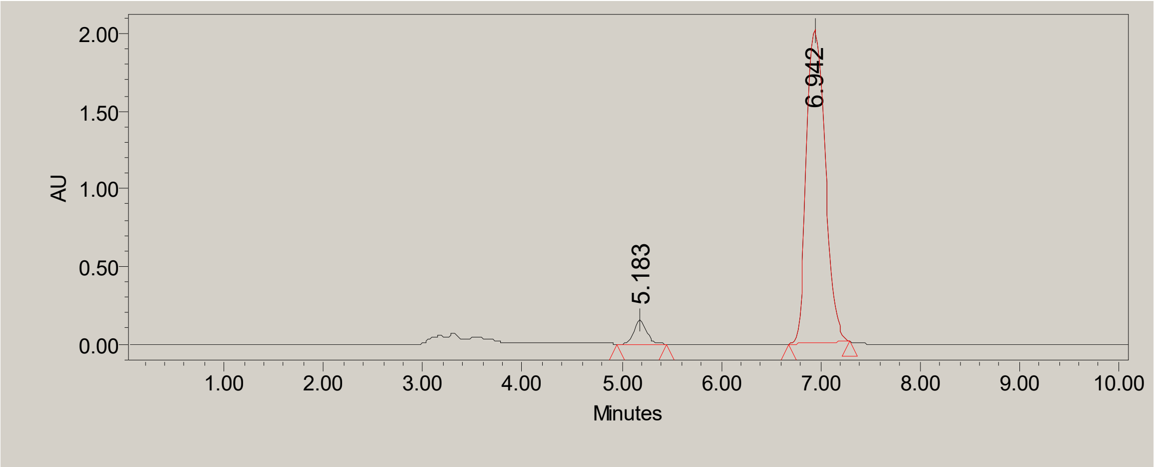

	Name	Retention Time	Area	% Area	Height	Int Type	Peak Type
1		5.183	1353926	4.83	154699	bb	Unknown
2		6.942	26669541	95.17	2015777	bb	Unknown

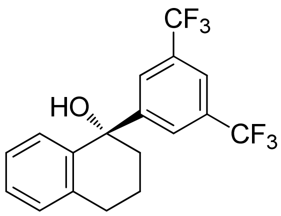

(*S*)-1-(3,5-Bis(trifluoromethyl)phenyl)-1,2,3,4-tetrahydronaphthalen-1-ol (2h)

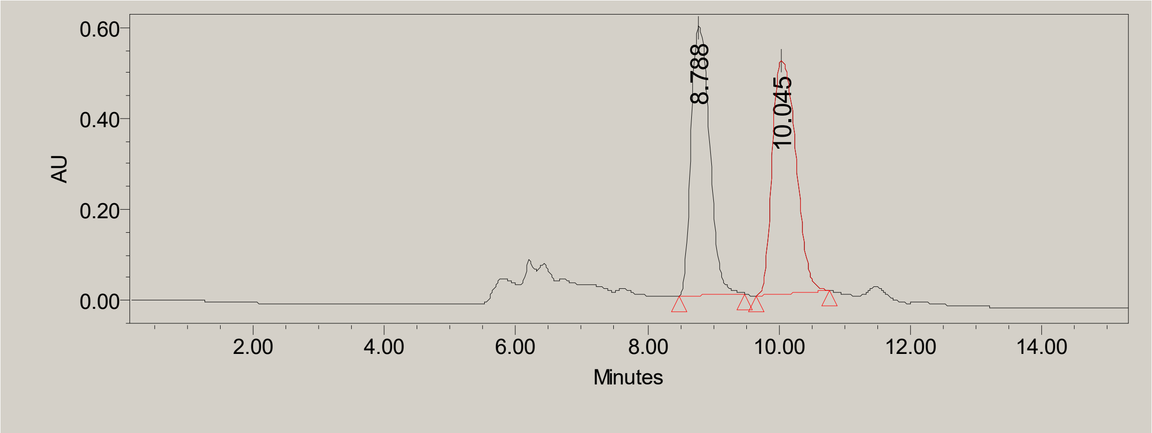

	Name	Retention Time	Area	% Area	Height	Int Type	Peak Type
1		8.788	11628585	48.24	591291	bb	Unknown
2		10.045	12478281	51.76	506647	bb	Unknown

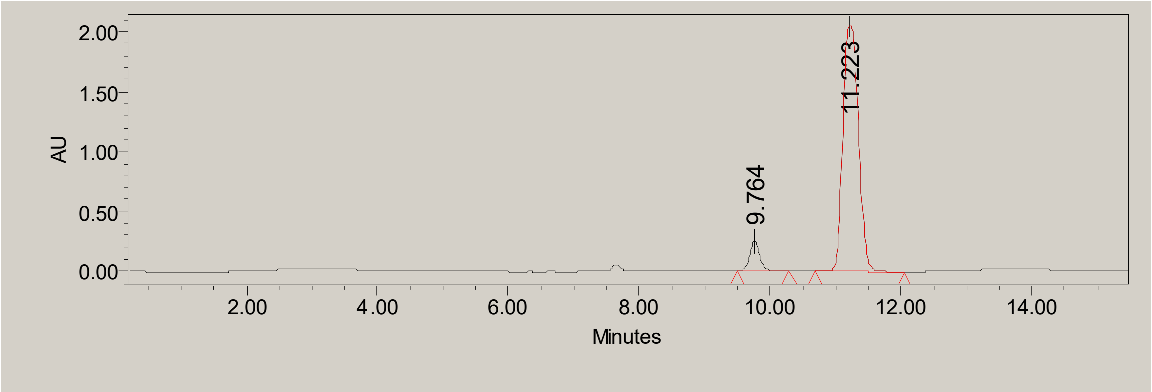

	Name	Retention Time	Area	% Area	Height	Int Type	Peak Type
1		9.764	2531588	6.96	243663	bb	Unknown
2		11.223	33862381	93.04	2055586	bb	Unknown

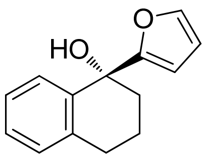

(*R*)-1-(Furan-2-yl)-1,2,3,4-tetrahydronaphthalen-1-ol (2i)

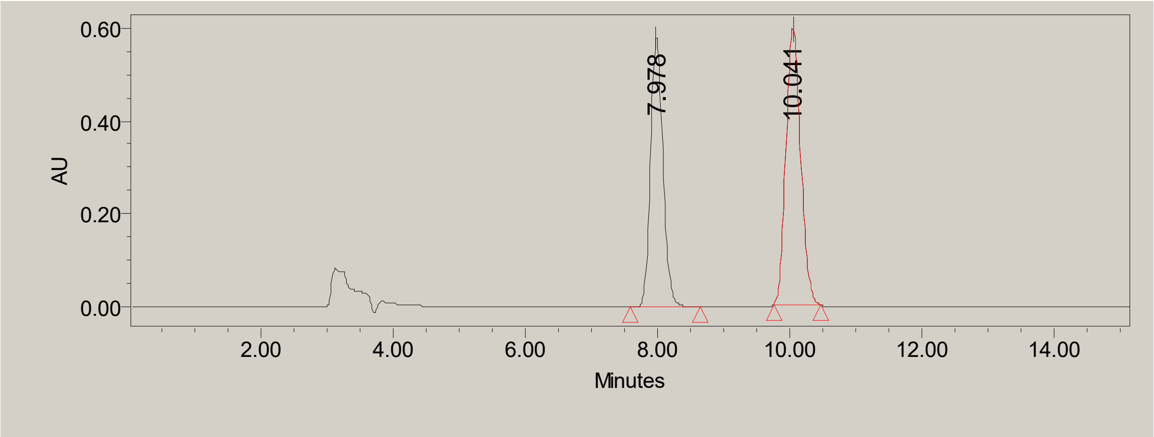

	Name	Retention Time	Area	% Area	Height	Int Type	Peak Type
1		7.978	7529468	48.32	583186	bb	Unknown
2		10.041	8052134	51.68	560857	bb	Unknown

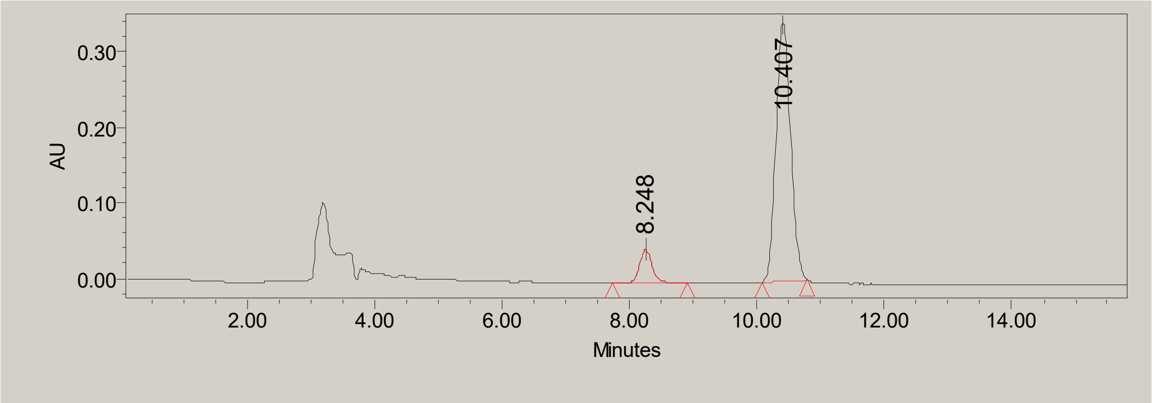

	Name	Retention Time	Area	% Area	Height	Int Type	Peak Type
1		8.248	591824	10.68	43882	bb	Unknown
2		10.407	4950348	89.32	327360	bb	Unknown

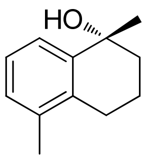

(*R*)-1,5-Dimethyl-1,2,3,4-tetrahydronaphthalen-1-ol (2j)

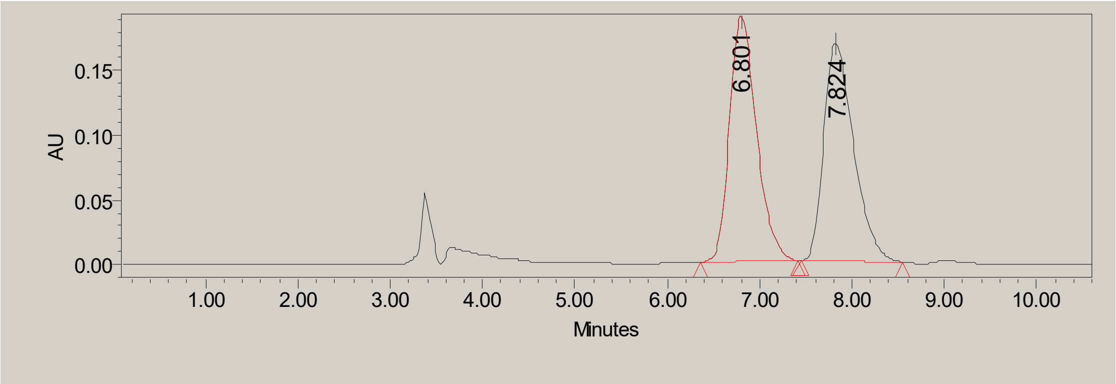

	Name	Retention Time	Area	% Area	Height	Int Type	Peak Type
1		6.801	3847974	50.34	189483	bb	Unknown
2		7.824	3795404	49.66	168236	bb	Unknown

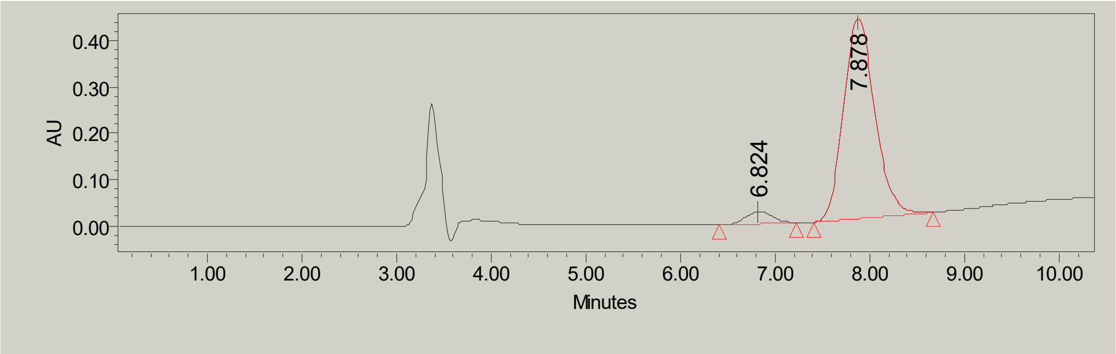

	Name	Retention Time	Area	% Area	Height	Int Type	Peak Type
1		6.824	514100	4.93	25646	bb	Unknown
2		7.878	9911947	95.07	430828	bb	Unknown

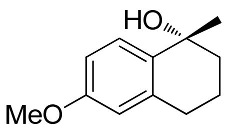

(*R*)-6-Methoxy-1-methyl-1,2,3,4-tetrahydronaphthalen-1-ol (2k)

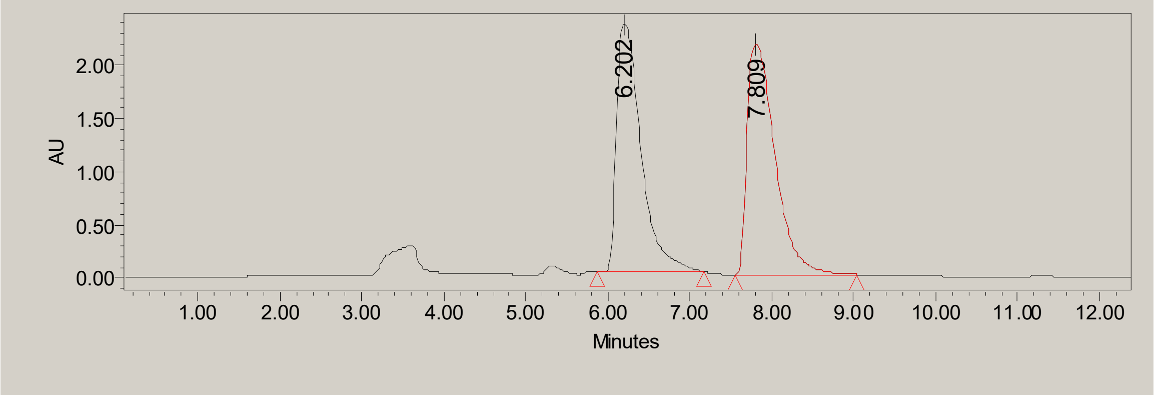

	Name	Retention Time	Area	% Area	Height	Int Type	Peak Type
1		6.202	48895820	48.59	2335090	bb	Unknown
2		7.809	51730094	51.41	2173704	bb	Unknown

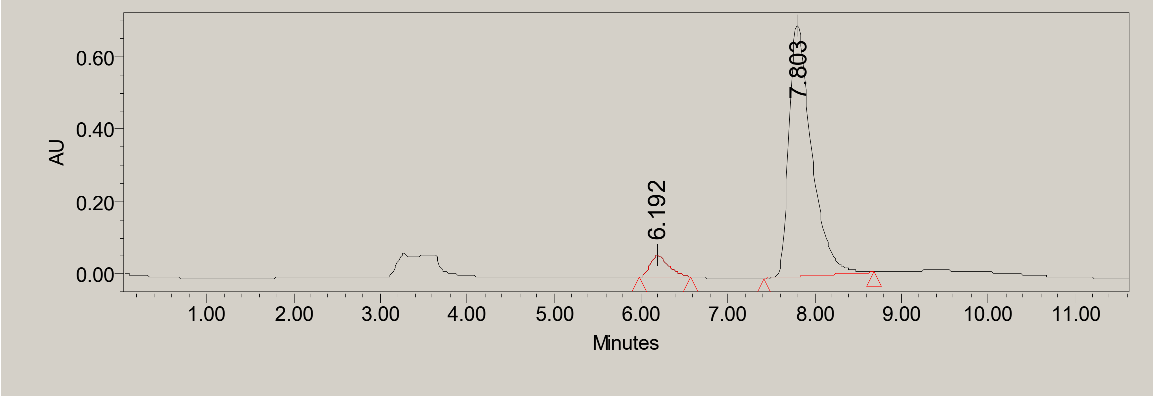

	Name	Retention Time	Area	% Area	Height	Int Type	Peak Type
1		6.192	813848	6.07	58472	bb	Unknown
2		7.803	12600924	93.93	693669	bb	Unknown

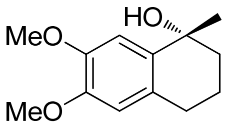

(*R*)-6,7-Dimethoxy-1-methyl-1,2,3,4-tetrahydronaphthalen-1-ol (2l)

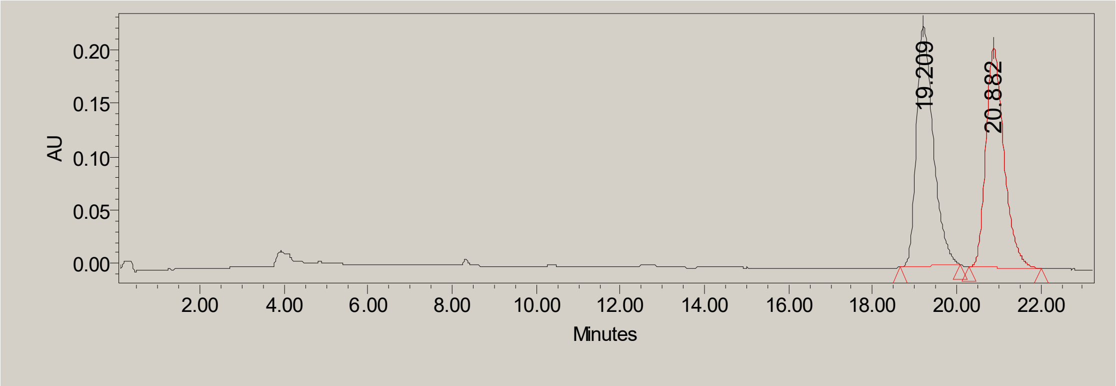

	Name	Retention Time	Area	% Area	Height	Int Type	Peak Type
1		19.209	6183040	50.04	224492	bb	Unknown
2		20.882	6173105	49.96	204407	bb	Unknown

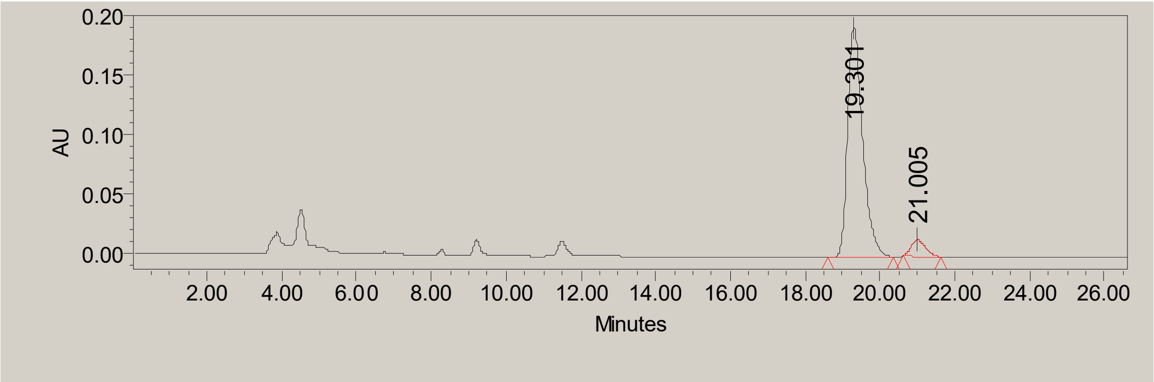

	Name	Retention Time	Area	% Area	Height	Int Type	Peak Type
1		19.301	5431089	93.93	194322	bb	Unknown
2		21.005	351189	6.07	13350	bb	Unknown

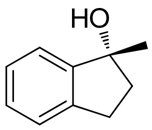

(*R*)-1-Methyl-2,3-dihydro-1H-inden-1-ol (2m)

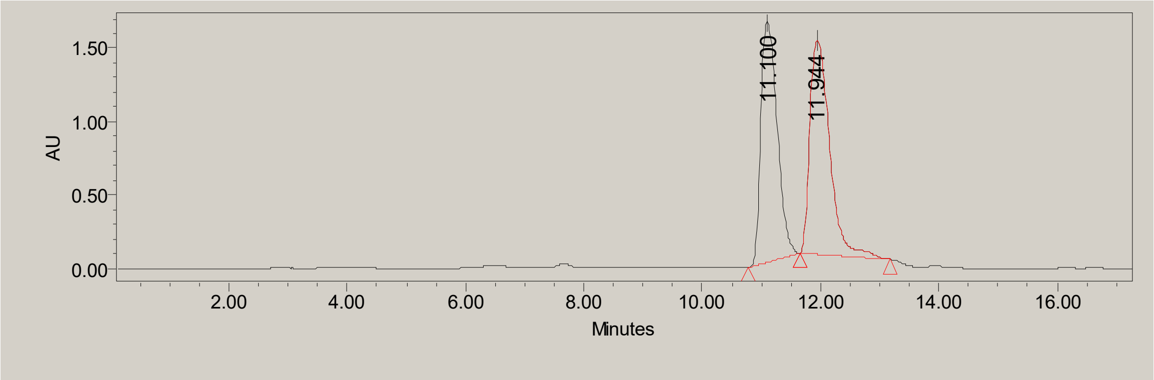

	Name	Retention Time	Area	% Area	Height	Int Type	Peak Type
1		11.100	31360518	48.12	1640168	bb	Unknown
2		11.944	33813843	51.88	1455984	bb	Unknown

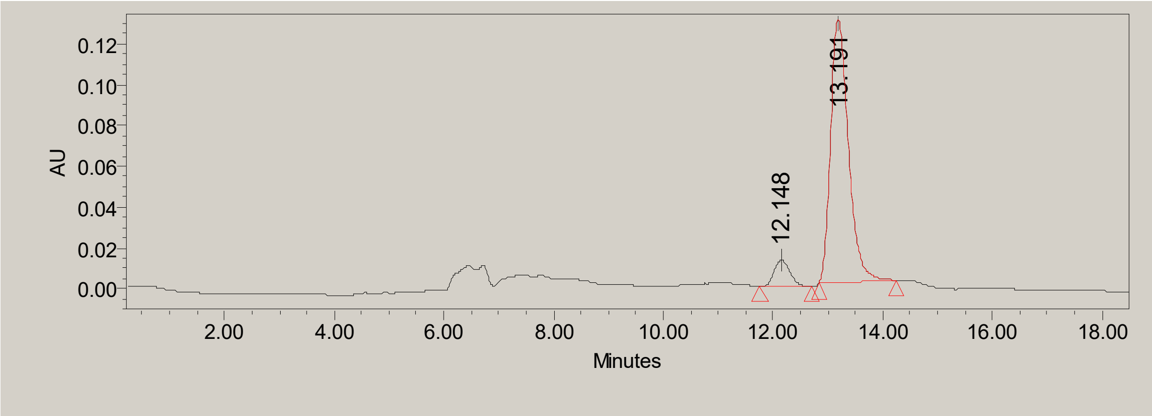

	Name	Retention Time	Area	% Area	Height	Int Type	Peak Type
1		12.148	250801	8.91	12774	bb	Unknown
2		13.191	2564291	91.09	121235	bb	Unknown
